# Area Law for the Entanglement Entropy of Free Fermions in Nonrandom Ergodic Field

**DOI:** 10.3390/e28050509

**Published:** 2026-05-01

**Authors:** Leonid Pastur, Mira Shamis

**Affiliations:** 1 Department of Mathematics, King’s College London, Strand, London WC2R 2LS, UK; 2 Theoretical Department, B. Verkin Institute for Low Temperature Physics and Engineering, 47 Nauky Avenue, 61103 Kharkiv, Ukraine; 3 School of Mathematical Sciences, Queen Mary University of London, Mile End Road, London E1 4NS, UK; m.shamis@qmul.ac.uk; 4 School of Mathematical Sciences, Holon Institute of Technology, 52 Golomb St., P.O. Box 305, Holon 5810201, Israel

**Keywords:** entanglement entropy, free fermions, area law, nonrandom ergodic fields

## Abstract

The paper deals with the asymptotic behavior of a widely used correlation characteristic in large quantum systems. The correlation is quantum entanglement, the characteristic is entanglement entropy, and the system is an ideal gas of lattice fermions. If the one-body Hamiltonian of fermions is an ergodic finite difference operator with an exponentially decaying spectral projection, then the large-block form of the entanglement entropy is the so-called area law. However, the only class of one-body Hamiltonians for which this spectral condition was verified consists of discrete Schrödinger operators with random potential. In this paper, we prove the area law for several classes of Schrödinger operators whose potentials are ergodic but not random. We begin with quasiperiodic and limit-periodic operators and then move to a highly non-trivial case of potentials generated by subshifts of finite type. These arose in the theory of dynamical systems when studying chaotic phenomena. The corresponding asymptotic study requires involved spectral analysis, which therefore constitutes the bulk of the paper. Specifically, we prove uniform localisation of the eigenfunctions for the Maryland model and exponential decay of the eigenfunction correlator for various models. We believe these properties are of significant independent interest.

## 1. Introduction

Quantum entanglement is one of the basic properties of quantum systems. Introduced by Einstein, Rosen, and Podolsky in 1935 to prove the incompleteness of quantum mechanics and immediately identified by Schrödinger as an important form of quantum correlations, quantum entanglement is now the subject of active research in both fundamental and applied fields, see, e.g., review works [1,2,3,4,5,6,7] and references therein.

One of the widely used quantitative characteristics of quantum entanglement between two subsystems of a quantum system is the entanglement entropy. Much of its relevant studies, which are also of importance for quantum statistical mechanics, quantum gravity, and quantum computing, deal with the analysis of the asymptotic behavior of the entanglement entropy of a sufficiently large (mesoscopic) subsystem confined to a finite block Λ of a macroscopically large quantum system occupying the whole $Z^{d}$ or $R^{d}$.

As a result of numerous theoretical, experimental, and numerical works of recent decades, it was found on various levels of rigor that the following two basic asymptotic forms of the entanglement entropy $S_{\Lambda }$ of a block Λ of linear size *L* are valid for a wide class of quantum systems.

- Area Law:

(1) $$S_{\Lambda }=C^{\prime }L^{d-1}\left(1+o\left(1\right)\right),\;L\to \infty ,$$

if the system ground state is not critical (no quantum phase transition) or/and if there is a spectral gap between the ground state and the rest of the spectrum;

- Enhanced Area Law:

(2) $$S_{\Lambda }=C^{\prime \prime }L^{d-1}\log L\left(1+o\left(1\right)\right),\;L\to \infty ,$$

if the system ground state is critical (a quantum phase transition is the case).

For the sake of completeness, we will also mention one more asymptotic form of entropy, which is now known as the

- Volume law

$$S_{\Lambda }=C^{\prime \prime \prime }L^{d}\left(1+o\left(1\right)\right),\;L\to \infty ,$$

which dates back to the origin of quantum statistical mechanics and is the case if the system is either in a mixed state, say, the Gibbs state of non-zero temperature, or in a pure but sufficiently highly excited state.

The rigorous proof of these asymptotic formulae, especially (1) and (2), proved to be quite nontrivial in the general case of multidimensional interacting quantum systems. This is why considerable attention has been paid to a simple yet nontrivial system of free fermions. The system, known since the late 1920s as a fairly adequate model of metals, has a number of properties that make it quite interesting in modern physics and related fields. The system is completely characterized by its one-body Hamiltonian *H*, a selfadjoint operator in $\ell^{2}\left(Z^{d}\right)$ or $L^{2}\left(R^{d}\right)$.

We will confine ourselves to the lattice case, i.e., to $Z^{d}$ as the space where the system lives. Then, the one-body Hamiltonian is a selfadjoint operator in $\ell^{2}\left(Z^{d}\right)$

(3) $$H={\left\{H\left(m,n\right)\right\}}_{m,n\in Z^{d}},\;\bar{H(m,n)}=H\left(n,m\right),$$

and one of the most important cases is where *H* is a discrete Schrödinger operator

(4) $$\begin{array}{cc} \left(H\psi )(n\right) & =(\Delta \psi )(n)+(V\psi )(n)\\ \; & =\sum_{|n-m|=1}\psi \left(m\right)+V\left(n\right)\psi \left(n\right),\;n,m\in Z^{d}, \end{array}$$

which is an archetype model of the field.

Denote by σ(H) the spectrum of *H* and by $\varepsilon_{-}$ and $\varepsilon_{+}$ the extreme endpoints of σ(H), so that

(5) $$\sigma \left(H\right)\subset \left[\varepsilon_{-},\varepsilon_{+}\right]=:I\left(H\right).$$

Let $E_{H}\left(d\lambda \right)$ be the resolution of the identity of *H*. Introduce the Fermi projection

(6) $$P\left(\varepsilon_{F}\right)=E_{H}\left(\left(\varepsilon_{-},\varepsilon_{F}\right]\right)=\chi_{\left(\varepsilon_{-},\varepsilon_{F}\right]}\left(H\right),$$

where $\varepsilon_{F}$ is a parameter of the system, called the Fermi energy (the ground state energy per particle for free fermions), and $\chi_{\left(\cdot ,\cdot \right]}\left(\cdot \right)$ is the indicator function.

Then the entanglement entropy of the finite block $\Lambda \subset Z^{d}$ of free fermions is [8,9]

(7) $$S_{\Lambda }\left(\varepsilon_{F}\right)={\mathrm{Tr}}_{\Lambda }h\left(P_{\Lambda }\left(\varepsilon_{F}\right)\right),$$

where ${\mathrm{Tr}}_{\Lambda }$ denotes the (restricted) trace in $\ell^{2}\left(\Lambda \right)$,

(8) $$P_{\Lambda }\left(\varepsilon_{F}\right)=\chi_{\Lambda }P\left(\varepsilon_{F}\right)\chi_{\Lambda }\equiv P\left(\varepsilon_{F}\right){|}_{\Lambda }$$

is the restriction of $P(\varepsilon_{F})$ of (6) to $\ell^{2}\left(\Lambda \right)$,

(9) $$\chi_{\Lambda }:\ell^{2}\left(Z^{d}\right)\to \ell^{2}\left(\Lambda \right)$$

is the coordinate projection, and

(10) h(x)=−xlogx−(1−x)log(1−x),x∈[0,1]

is the Shannon binary entropy.

Note that if $\varepsilon_{F}\notin I\left(H\right)$ of (5), then $S_{\Lambda }\left(\varepsilon_{F}\right)=0$. Thus, from now forward, we are assuming that

(11) $$\varepsilon_{F}\in I\left(H\right),$$

i.e., $\varepsilon_{F}$ belongs either to the spectrum of *H* or to its internal gap.

To expect the regular asymptotic behavior of the entanglement entropy, one has to assume a certain “homogeneity” of the one-body Hamiltonian. This is why we will consider the class of the so-called ergodic operators that possess this important property. The class is defined as follows.

Let (Ω,F,P) be a probability space equipped with a measure-preserving *d*-dimensional group of ergodic transformations ${\left\{T^{n}:\Omega \to \Omega \right\}}_{n\in Z^{d}}$. Then, a *d*-dimensional ergodic operator acting in $\ell^{2}\left(Z^{d}\right)$ is an operator-valued random variable assuming values in selfadjoint operators such that (see (3))

(12) $$H_{\omega }={\left\{H\left(\omega ,m,n\right)\right\}}_{m,n\in Z^{d}},\;H\left(T^{k}\omega ,m,n\right)=H\left(\omega ,m+k,n+k\right),$$

see, e.g., Refs. [10,11] for spectral theory of this class of operators.

The simplest but quite important subclass of ergodic operators in $\ell^{2}\left(Z^{d}\right)$ is discrete convolution operators, where Ω={∅} and

(13) $$H={\left\{H\left(m-n\right)\right\}}_{m,n\in Z^{d}},\sum_{m\in Z^{d}}\left|H\left(m\right)\right|<\infty .$$

It was shown in a series of works (see [12,13,14,15,16] and references therein), that for a wide class of translation invariant pseudodifferential operators (including (13)), the corresponding entanglement entropy obeys the Enhanced Area Law (2) if the Fermi energy $\varepsilon_{F}$ of (6)–(8) belongs to the (absolutely continuous) spectrum $\sigma \left(H\right)=\sigma_{ac}\left(H\right)$ of *H*.

This should be compared with the “opposite” case of random ergodic operators, where $\Omega =R^{Z}$, (e.g., the discrete Schrödinger operators with an independent identically distributed (i.i.d.) potential [10,11]), where the Area Law (1) for the expectation $E\{S_{\Lambda }\left(\varepsilon_{F}\right)\}$ and even for the overwhelming majority of realization holds for $\varepsilon_{F}$ belonging to a certain part of the pure point spectrum $\sigma_{pp}\left(H\right)\subset \sigma \left(H\right)$, which is due to the so-called “strong” Anderson localisation [17,18]. Recall that the spectrum of an ergodic operator and all its components are deterministic, i.e., are independent of ω with probability 1.

An interesting observation that follows from the above is that the asymptotic behavior of the entanglement entropy is closely related to the spectral type of the corresponding one-body operator. Namely, for convolution operators (translation invariant case), where the spectrum is purely absolutely continuous and the generalized eigenfunctions are plane waves, we have the Enhanced Area Law (2), while for random operators (disordered case), where the spectrum is pure point and eigenfunctions are exponentially decaying, we have the Area Law (1). Here, it is appropriate to recall a result of [19], according to which the exponential decay of correlation functions in one-dimensional quantum systems implies the Area Law for the entanglement entropy of their states under certain additional conditions. However, this observation cannot be true as formulated, since, for example, there exist random Schrödinger operators with a purely point spectrum that exhibit the Enhanced Area Law (2) for isolated values of the Fermi energy [20]. This is because the asymptotic form of the entanglement entropy is determined by the behavior at $\varepsilon_{F}$ (and, possibly, in Λ-infinitesimal neighborhoods of $\varepsilon_{F}$) of the entries ${\left\{P\left(m,n\right)\right\}}_{m,n\in Z^{d}}$ of the Fermi projection as |m−n|→∞, while the spectral type is determined by the spatial behavior of the matrix on Λ-independent intervals (see, e.g., (40) and (42)). A similar “sensitivity” is also known for some transport characteristics of corresponding disordered systems with the same single-particle Hamiltonian (moments of the position operator, d.c. conductivity, etc.), see [21,22] and references therein.

Nevertheless, the observation allows us to expect that the Area Law holds for the classes of ergodic operators other than random ones, provided that they also exhibit sufficiently “strong” Anderson localisation. In particular, these are finite difference operators with quasi-periodic potentials, defined on an orbit of an irrational winding on the torus, or with potentials that are functions defined on an orbit of more complex finite-dimensional dynamical systems, including the doubling map and the famous Arnold’s cat map [23,24].

The goal of this paper is to prove the Area Law for these classes of ergodic operators. The paper is organized as follows. In Section 2, we present our results on the validity of the Area Law for free lattice fermions whose one-body Hamiltonian is a finite-difference operator in $\ell^{2}\left(Z^{d}\right)$ with dynamically generated coefficients. Specifically, these are self-adjoint ergodic operators that are the sums of a convolution operator (13) (the discrete Laplacian in the case of Schrödinger operator (4)) and a dynamically generated potential, see (14), (15) and (17) below. Theorems 1 and 2 treat multidimensional and one-dimensional quasiperiodic and limit-periodic cases. Theorem 4 treats the case where the one-body Hamiltonian is the one-dimensional Schrödinger operator with potentials generated by a subshift of finite type, a dynamical system that is much more complicated than the irrational shift on T=R/Z, which generates quasi-periodic potentials.

All of these operators have a purely point component of spectrum. However, our proof, based on the results of [17], makes significant use of some form of exponential bounds on the spectral (Fermi) projection of the operator in question, i.e., a considerably stronger property. In certain cases, this property is established and we just use relevant results, sometimes transforming them into a form applicable to our problem. There are, however, several important cases where the existing spectral theory has to be considerably developed. The first is the case of the archetype Maryland model, both multi-dimensional and one-dimensional, where we have to prove the property of uniform localisation of eigenfunctions, presented in Theorem 3. The second important case is the Schrödinger operator whose potentials are generated by the subshift of finite type. Here, we have to prove the exponential decay of the eigenfunction correlator, presented in Theorem 5. This includes a useful criterion for the exponential decay of the eigenfunction correlator for ergodic bounded one-dimensional discrete Schrödinger operators in terms of control over the “bad” sets of spectral parameters of the operator restriction to large but distant boxes, see Lemma 1. We believe that these spectral results are of independent interest.

## 2. Results and Comments

In this section we formulate our main results and make certain comments on their meaning and proof.

All results correspond to the one-body operator (cf. (3))

(14) $$\begin{array}{cc} \; & H_{\omega }=W+V_{\omega }={\left\{H\left(\omega ,m,n\right)\right\}}_{m,n\in Z^{d}},\\ \; & \;H(\omega ,m,n)=W(m-n)+V(\omega ,n)\delta (m-n), \end{array}$$

where

(15) $$\begin{array}{cc} \; & W\left(-n\right)=\bar{W(n)},\;\left|W\left(n\right)\right|\leq We^{-\rho |n|},\\ \; & \;W<\infty ,\;\rho >0,\;|n|=|n_{1}\left|+\cdots +\right|n_{d}|, \end{array}$$

and its particular case—Schrödinger operator (4), where *W* is the *d*-dimensional discrete Laplacian

(16) $$W\left(n\right)=\delta_{n+e_{j}}+\delta_{n-e_{j}},\;n\in Z^{d},\;j=1,\ldots ,d,$$

and ${\left\{e_{j}\right\}}_{j=1}^{d}$ is the canonical basis in $Z^{d}$.

Since $H_{\omega }$ is ergodic (see (3)), its potential $V_{\omega }{=\{V\left(n,\omega )\right\}}_{n\in Z^{d}}$ in (14) is an ergodic field in $Z^{d}$. Namely, it is given by a measurable (sample) function v:Ω→R on a probability space (Ω,F,P) equipped with a measure preserving and ergodic group of automorphisms ${\left\{T^{n}:\;\Omega \to \Omega \right\}}_{n\in Z^{d}}$, so that

(17) $$V_{\omega }={\left\{V\left(\omega ,n\right)\right\}}_{n\in Z^{d}},\;V\left(\omega ,n\right)=v\left(T^{n}\omega \right),\;n\in Z^{d},\;\omega \in \Omega .$$

We will also assume in what follows that the block in (7)–(9) is a cube

$$\Lambda ={\left[-M,M\right]}^{d}\subset Z^{d},\;2M+1=L.$$

### 2.1. Results

We present here the results on the validity of the Area Law (1) for several interesting classes of ergodic operators. Recall that the only class of these operators for which the Area Law has been rigorously established is of the Schrödinger operators with independent identically distributed random potentials whose probability law possesses a certain amount of smoothness [17].

We will begin with results valid in any dimension d≥1. We denote by $T^{d}=R^{d}/Z^{d}$ the *d*-dimensional torus for any d≥1, and by 〈·,·〉 the scalar product in $Z^{d}$.

**Theorem** **1.** *The expectation* $E\{S_{\Lambda }\left(\varepsilon_{F}\right)\}$ *of the entanglement entropy* (7)–(10) *of free lattice fermions obeys the Area Law* (1) *for the following ergodic one-body Hamiltonians acting in* $\ell^{2}\left(Z^{d}\right),\;d\geq 1$*:*

*(i)*  *The multidimensional Maryland model: the operator* (14) *and* (15) *with the potential*
  (18) $$V\left(\omega ,n\right)=g\;\tan\pi \left(\omega +\langle n,\alpha \rangle \right),\;n\in Z^{d},\;\omega \in T=\Omega ,\;\;g\neq 0,$$
  *where $\alpha \in T^{d}$ satisfies the multidimensional Diophantine condition*
  (19) $${\left||\langle n,\alpha \rangle |\right|}_{d}\geq c{\left|n\right|}^{-\tau },\;c>0,\tau >0,\;n\in Z^{d}\setminus \left\{0\right\},$$
  *with ${∥\langle n,\alpha \rangle ∥}_{d}=\mathrm{dist}\left(\langle n,\alpha \rangle ,Z\right)$ and the Fermi energy $\varepsilon_{F}\in R$ (cf. Theorem 2 (i) about the* 1*-d case below).*
*(ii)*  *The Schrödinger operators (4) with the potential*
  (20) $$V\left(\omega ,n\right)=g\;v\left(\omega +\langle n,\alpha \rangle \right),\;n\in Z^{d},\;\omega \in T,$$
  *where g>0 is sufficiently large, v:R→R is* 1*-periodic function with the ξ-Hölder monotone property*
  (21) $$v\left(y\right)-v\left(x\right)\geq {\left(y-x\right)}^{\xi },\;\xi \geq 1,\;0\leq x\leq y<1,$$
  *$\alpha \in T^{d}$ satisfies a weak Diophantine condition (cf.* (19) *and* (24)*)*
  (22) $$\Omega_{\rho ,\mu }=\left\{\alpha \in T^{d}\;:\;{∥\langle n,\alpha \rangle ∥}_{d}\geq \exp\{-{\rho |n|}^{\frac{1}{1+\mu }}\},n\neq 0\right\},$$
  *with ρ,μ>0, ${∥\langle n,\alpha \rangle ∥}_{d}=\mathrm{dist}\left(\langle n,\alpha \rangle ,Z\right)$, and the Fermi energy is*
  $$\varepsilon_{F}\in I\left(H\right),$$
  *where I(H) is defined in* (5) *(cf. Theorem 2 (iii)).*
*(iii)*  *The operator* (14) *with* ${\left\{W\left(n\right)\right\}}_{n\in Z^{d}}$ *satisfying* (15)*, and with the multidimensional analog*
  (23) $$V\left(\omega ,n\right)=g\cos2\pi \left(\omega +\langle n,\alpha \rangle \right),\;\;n\in Z^{d},\;\omega \in T,$$
  *of the almost Mathieu potential (32), where g is large enough, $\alpha \in DC_{d}$ with*
  (24) $$DC_{d}=\underset{\kappa >0,\tau >d-1}{⋃}\left\{\alpha \in T^{d}:\underset{j\in Z}{\inf}\left|\langle n,\alpha \rangle -j\right|>\kappa {\left|n\right|}^{-\tau },\;\;n\in Z^{d}\setminus \left\{0\right\}\right\},$$
  *(cf.* (19)*), and with the Fermi energy $\varepsilon_{F}\in I\left(H\right)$, where I(H) is defined in* (5)*, see also* (11)*.*
*(iv)*  *The Schrödinger operators (4) with limit-periodic potentials*
  (25) $$V_{\omega }\left(n\right)=f\left(T^{n}\omega \right),\;n\in Z^{d},\;\omega \in \Omega ,$$
  *where* Ω *is a Cantor group that admits a minimal $Z^{d}$ action T by translations (see Definition 3), and f∈C(Ω,R) (the existence of such* Ω *and f is proved in [25], see Proposition 3 below), where C(Ω,R) is the space of continuous functions f:Ω→R, and the Fermi energy $\varepsilon_{F}\in I\left(H\right)$, where I(H) is defined in* (5)*, see also* (11)*.*

**Remark** **1.** *(i) There is a one-dimensional version of Theorem 1 (iv). It was considered in [26] where an explicit condition on the Cantor group* Ω *is formulated.*

*(ii) Examples of the corresponding ergodic limit-periodic potentials can be obtained by using the techniques developed in [25,26,27,28] and limit-periodic potentials found in [29].*

We will present now certain one-dimensional results on the validity of the Area Law that are either more general or stronger than their multidimensional counterparts given in the above theorem. The results are mostly for the one-dimensional Schrödinger operator (cf. (4) and (14))

(26) (Hψ)(n)=ψ(n+1)+ψ(n−1)+(Vψ)(n),n∈Z,

with quasi-periodic and limit-periodic potentials.

We have already seen that the spectral properties of quasiperiodic operators depend strongly on the arithmetic properties of the potential frequencies. Here is a natural quantifier of their “irrationality”

(27) $$\beta \left(\alpha \right)=\lim\underset{n\to \infty }{\sup}\frac{-{\log||n\alpha ||}_{T}}{n}=\lim\underset{n\to \infty }{\sup}\frac{\log q_{n+1}}{q_{n}},$$

where ${\left\{q_{n}\right\}}_{n\in N}$ are denominators of the continuous fraction expansion of $\alpha =\lim_{n\to \infty }p_{n}/q_{n}$ and $\left|\right|\cdot {\left|\right|}_{T}={\mathrm{dist}}_{H}\left(\cdot ,Z\right)$ (the Hausdorff distance).

The frequencies α for which β(α)=0 are “generalized” Diophantine, cf. (19), and those with 0<β(α)≤∞ are Liouvillian.

**Theorem** **2.** *The expectation $E\{S_{\Lambda }\left(\varepsilon_{F}\right)\}$ of entanglement entropy (7)–(10) of free lattice fermions with d=1 obeys the Area Law (1) for the following one-body Hamiltonians:*

*(i)*  *The one-dimensional Maryland model given by* (14)*–*(16) *for d=1, i.e., the one-dimensional discrete Schrödinger operator (26) with the potential (cf.* (18)*)*
  (28) V(ω,n)=gtanπ(ω+nα),ω∈T,α∈T,g≠0,
*and with the Fermi energy (6) $\varepsilon_{F}\in \sigma_{-}\left(H_{\omega }\right):=\left(-\infty ,-\varepsilon_{0}\right)$, where $\varepsilon_{0}\geq 0$ solves the equation*
(29) $$\begin{array}{c} \beta \left(\alpha \right)=\gamma \left(\varepsilon_{0},g\right), \end{array}$$
  *with β(α) given in (27) and γ(λ,g)≥γ(0,g)>0 being the Lyapunov exponent of the corresponding finite-difference equation of the second order* (68) *(see* (87) *for the general definition of the Lyapunov exponent, and* (69)*–*(71) *for the form of γ(λ,g) in the one-dimensional Maryland model). If γ(0,g)≥β(α), then we have $\varepsilon_{F}\in R$.*
*(ii)*  *The one-dimensional Schrödinger operator (26) with potential*
(30) V(ω,n)=gv(ω+nα),ω∈T,α∈T,
  *where v:R→R is* 1*-periodic, continuous on [0,1), v(0)=0,v(1−0)=1, and Lipschitz monotone (cf.* (21)*)*
  (31) $$\begin{array}{ccc} & & a_{-}\left(y-x\right)\leq v\left(y\right)-v\left(x\right)\leq a_{+}\left(y-x\right),\;\;\\ & & \;0\leq x\leq y<1,\;a_{\pm }>0, \end{array}$$
  *α satisfies the Diophantine condition (cf.* (19)*)*
  $${∥n\alpha ∥>C|n|}^{-\tau },\;\;n\in Z,\;\tau >0,$$
*where ∥·∥=min({·},{1−·}), g>0 is large enough, and*
$$\varepsilon_{F}\in I\left(H\right)\subset \left[-2,2+g\right],$$
  *where I(H) is defined in* (5)*, see also* (11)*.*
*(iii)*  *The supercritical almost Mathieu operator: the one-dimensional discrete Schrödinger operator (26) with potential*
(32) V(ω,n)=2gcos2π(ω+nα),ω∈T,|g|>1,
*where α∈T is such that 0≤β(α)<β for some β>0, β(α) is defined by (27), and the Fermi energy satisfies*
$$\varepsilon_{F}\in I\left(H\right)\subset \left[-(2+2|g|),(2+2|g|)\right]$$
  *where I(H) is defined in* (5)*, see also (11).*

The crucial ingredient of the proof of Theorems 1 (i) and 2 (i) is the following assertion of independent interest, according to which the eigenfunctions of the multi- and one-dimensional Maryland model are uniformly localised. This result provides a first example of uniformly localised eigenfunctions for an *unbounded* operator on the whole (infinite) spectrum. In the one-dimensional case, the spectrum of the corresponding operator (the one-dimensional Schrödinger operator (26) with potential (28)) is described by similar formulae. However, since, in this case, the operator *W* in (14) and (15) is the one-dimensional discrete Laplacian, an important part of the proof of the multidimensional estimate (33) can be made explicit. thereby leading to a stronger one-dimensional version.

**Theorem** **3.**

*(i)*  *Let d>1, and let $H_{\omega }$ be the multidimensional Maryland model, the operator* (14) *and* (15) *with the potential* (18)*, where α satisfies the multidimensional Diophantine condition* (19)*. Then the spectrum of $H_{\omega }$ occupies the whole R, is pure point and of multiplicity 1 (see, e.g., Sections 18.A–18.B in [11]). If ${\left\{\lambda_{l}\left(\omega \right)\right\}}_{l\in Z^{d}}$ are its eigenvalues and ${\left\{\psi_{l}\left(\omega \right)\right\}}_{l\in Z^{d}}$, $\psi_{l}\left(\omega \right)={\left\{\psi_{l}\left(\omega ,n\right)\right\}}_{n\in Z^{d}}$ are the corresponding eigenfunctions of $H_{\omega }$, then there exist C<∞ and c>0 independent of λ∈R and of $n,l\in Z^{d}$ such that*
  (33) $$|\psi_{l}\left(\lambda_{l},n\right)|\leq Ce^{-c|n-l|},\;for\;any\;n,l\in Z^{d},$$
  *i.e., the operators have uniformly localised eigenfunctions* (37)*.*
*(ii)*  *Let $H_{\omega }$ be the* 1*-dimensional Maryland model* (28)*, γ(λ,g)≥γ(0,g)>0 be its Lyapunov exponent, and $\varepsilon_{0}>0$ be the solution of the equation $\beta \left(\alpha \right)=\gamma \left(\varepsilon_{0},g\right)$ with β(α) of* (27)*. Then the spectrum of $H_{\omega }$ is pure point on $\sigma_{\pm }\left(H_{\omega }\right):=\left\{\pm \lambda \geq \varepsilon_{0}>0\right\}$, and if $\varepsilon_{0}=0$, then $\sigma_{pp}\left(H_{\omega }\right)=\sigma_{-}\left(H_{\omega }\right)\cup \sigma_{+}\left(H_{\omega }\right)=R,$ see, e.g., Section 18.C in [11].*
*If ${\left\{\lambda_{l}\left(\omega \right)\right\}}_{l\in Z}$ are its eigenvalues and ${\left\{\psi_{l}\left(\omega \right)\right\}}_{l\in Z}$, $\psi_{l}\left(\omega \right)={\left\{\psi_{l}\left(\omega ,n\right)\right\}}_{n\in Z}$ are the corresponding eigenfunctions of $H_{\omega }$, then there exist C<∞ and c>0 independent of λ∈R and of n,l∈Z such that*
(34) $$|\psi_{l}\left(\omega ,m\right)|\leq Ce^{-c|m-l|},\;m\in Z,\;\left|\lambda \right|>\varepsilon_{0},$$
  *with ω-independent parameters C≤∞,c>0, and $\varepsilon_{0}\geq 0$, i.e., the operators have uniformly localised eigenfunctions* (37)*.*

The description of eigenfunctions of the Maryland model (see (49)–(51)) provides the simplest and a quite explicit illustration of the notion of the localisation centers and the uniformly localised eigenfunctions. These spectral objects can be defined for any selfadjoint operator *H* acting on $\ell^{2}\left(Z^{d}\right)$. Namely, *H* is said to have *uniformly localised eigenfunctions* (ULE) if and only if *H* has a complete set

(35) $${\left\{\psi_{l}\right\}}_{l},\;\psi_{l}={\left\{\psi_{l}\left(m\right)\right\}}_{m\in Z^{d}},$$

of orthonormal eigenfunctions such that there exist constants C<∞,c>0, and $m_{l}\in Z^{d},\;l=1,2,\ldots$ providing the bound

(36) $$|\psi_{l}\left(m\right)|<Ce^{-c|m-m_{l}|},\;m\in Z^{d}.$$

Thus, the eigenfunctions are “localised about” points ${\left\{m_{l}\right\}}_{l}$ — the “localisation centers”, which can provide a convenient indexation of the eigenfunctions and the corresponding eigenvalues.

If $H_{\omega }$ is a self-adjoint *ergodic* operator, then we say that $H_{\omega }$ has the ULE if and only if it possesses with probability 1 a complete set of orthonormal eigenfunctions ${\left\{\psi_{l}\left(\omega \right)\right\}}_{l},\;\psi_{l}\left(\omega \right)={\left\{\psi_{l}\left(\omega ,m\right)\right\}}_{m\in Z^{d}}$ (cf. (35)) satisfying with the same probability the bound

(37) $$|\psi_{l}\left(\omega ,m\right)|<Ce^{-c|m-m_{l}\left(\omega \right)|},\;m\in Z^{d},$$

with ω-independent C<∞ and c>0 but possibly ω-dependent localisation centers ${\left\{m_{l}\right\}}_{l}$.

Note that the ULE is a quite special property. For instance, it is not the case for the Schrödinger operators with random potential (the Anderson model) in any dimension and for the almost Mathieu operator (32), see, e.g., Ref. [30]. On the other hand, the ULE is most explicit and useful in the Maryland model and certain other models with almost periodic potentials, see, e.g., Refs. [26,31].

**Remark** **2.** *In the context of this paper, the ULE property, if it provides one-to-one labeling of all eigenfunctions, leads directly and simply to the exponential bounds* (42) *and* (47) *for the Fermi projection and eigenfunction correlator. It suffices just to apply the argument used in Formulae* (77)*–*(79) *or in Appendix A proving* (42) *and* (47) *for the Maryland model. Hence, in this case, Criterion 1 or Criterion 2 yield the Area Law and the exponential dynamical localisation in expectation.*

Proofs of Theorems 1–3 are given in Section 3.

**Remark** **3.** *Using recent results of Jitomirskaya and Kachkovskiy (Corollary 5.14 in [32]), it is possible to obtain a stronger version of Theorem 2 (ii).*

In our next result, we consider one-dimensional discrete Schrödinger operators (26) with potentials generated by a subshift of finite type (see Section 5), a quite interesting class of hyperbolic dynamical systems exhibiting the so-called classical dynamical chaos, see, e.g., Ref. [24]. For these ergodic operators, the spectral localisation outside of a discrete set of energies and for a range of coupling constants was recently proven in [33,34].

**Theorem** **4.** *Let $\left(\Omega_{A},T\right)$ be a subshift of finite type and P be a T-ergodic measure that has the bounded distortion property (85). Assume that T has a fixed point on $\Omega_{A}$, and let $v:\Omega_{A}\to R$ be an α-Hölder continuous, 0<α≤1, that is globally bunched, non-constant, or locally constant (see Definition* 5*). Consider the one-dimensional Schrödinger operator (26) whose potential is generated by a subshift of finite type, $V_{\omega }\left(n\right)=v\left(T^{n}\omega \right)$.*

*Then there exists a bottom part $\left(\varepsilon_{-},\varepsilon_{0}\right]$ of I(H) of (5) such that if the Fermi energy $\varepsilon_{F}\in \left(\varepsilon_{-},\varepsilon_{0}\right]$, then the expectation of the entanglement entropy (7) of the free lattice fermions whose one-body Hamiltonian is $H_{\omega }$ obeys the Area Law (1).*

To prove Theorem 4 (see the proof at the end of Section 5), we have to substantially extend the existing spectral theory. We believe that this extension (see Theorem 5 below) is of independent interest for the spectral theory of this class of operators and completes the results of important recent works [33,34].

**Theorem** **5.** *Let $\left(\Omega_{A},T\right)$ be a subshift of finite type and P be a T-ergodic measure that has the bounded distortion property (85). Assume that T has a fixed point on $\Omega_{A}$, and let $v:\Omega_{A}\to R$ be an α-Hölder continuous, 0<α≤1, that is globally bunched, non-constant, or locally constant (see Definition* 5*). Consider the one-dimensional Schrödinger operator $H_{\omega }\;\left(26\right)$, whose potential is generated by a subshift of finite type, $V_{\omega }\left(n\right)=v\left(T^{n}\omega \right)$, and let I⊂R be a set where we have the uniformly positive Lyapunov exponent and the uniform large deviation-type estimate (see Definition* 6*). Then there exist C<∞,c>0 such that for any $m,n\in Z_{+}$, we have*

(38) $$E\left\{Q_{I}\left(m,n\right)\right\}\leq Ce^{-c|m-n|},$$

*where $Q_{I}$ is the eigenfunction correlator (defined below in (43)) corresponding to $H_{\omega }$. In particular, the operator $H_{\omega }$ exhibits the exponential dynamical localisation in expectation (48) on I.*

**Remark** **4.** *The existence of the compact set $I⊃\sigma (H_{\omega })$ on which we have uniformly positive Lyapunov exponent and the uniform large deviation-type estimate is guaranteed by [33,34] (see* (90) *and below), thereby providing the validity of Theorem 5. The examples of subshifts of finite type to keep in mind are as follows:*

- *The doubling map*
  $$T_{2}:T\to T,\;T_{2}\omega =2\omega \left(\mathrm{mod}\;1\right),\;\;\;$$
- *Arnold’s cat map*
  $$\begin{array}{cc} \; & \;T_{\mathrm{cat}}:T^{2}\to T^{2},\;\;T_{\mathrm{cat}}\left(\omega_{1},\omega_{2}\right)=\left(\left(2\omega_{1}+\omega_{2}\right)\left(\mathrm{mod}\;1\right),\left(\omega_{1}+\omega_{2}\right)\left(\mathrm{mod}\;1\right)\right), \end{array}$$

*where the corresponding invariant measures are the Lebesgue measures on the one- and two-dimensional tori, respectively.*

*In these cases, we define the potential by $V_{\omega }\left(n\right)=gv\left(T^{n}\omega \right)$ with $v:\Omega_{A}\to R$ satisfying the assumptions of Theorem 4. Then, there exists $g_{0}>0$ such that for all $0<g\leq g_{0}$, Theorems 4 and 5 hold true. Moreover, as pointed out in (Remark 2.18 in [34]), $g_{0}$ is not too small if α is not too small.*

An important ingredient of the proof of Theorem 5 is the following lemma of independent interest. It provides a general condition for “strong” localisation, more precisely, for exponential dynamical localisation in expectation (48) for a bounded ergodic one-dimensional discrete Schrödinger operator in terms of the absence of quantum-mechanical tunneling between the distant domains of the system. Various similar conditions were often used in the analysis of Anderson localisation since the pioneering paper by Fröhlich and Spencer [35].

Let $M={\left\{M\left(j,k\right)\right\}}_{j,k=-n}^{n}$ be a (2n+1)×(2n+1) matrix. Denote by

(39) $$\begin{array}{cc} \; & \;\mathrm{Bad}\;(M,\epsilon )=\\ \; & {\{\lambda \in R:\;\max(|\left(M-\lambda \right)}^{-1}{\left(0,n\right)|,|\left(M-\lambda \right)}^{-1}\left(0,-n\right)|)\geq \epsilon \}, \end{array}$$

where ${\left(M-\lambda \right)}^{-1}\left(k,l\right)$ is the (k,l) element of the resolvent matrix ${\left(M-\lambda \right)}^{-1}$, the set of the “badly”-controlled spectral parameters.

**Lemma** **1.** *Let H be a bounded ergodic one-dimensional discrete Schrödinger operator (26). Assume that for any $n\geq n_{0}$ and for any $\left|k|,|l\right|\leq n^{2}$ such that |k−l|≥10n and for some c>0*

$$\begin{array}{cc} \; & \left\{\cap_{i,j\in \{0,1\}}\mathrm{Bad}\;\left(H^{\left[k-n+i,k+n-j\right]},e^{-cn}\right)\right\}\\ \; & \;\cap \left\{\cap_{i,j\in \{0,1\}}\mathrm{Bad}\;\left(H^{\left[l-n+i,l+n-j\right]},e^{-cn}\right)\right\}=\emptyset , \end{array}$$

*where $H^{\left[m,p\right]}$ denotes the restriction of H to the interval [m,p]∩Z with Dirichlet boundary conditions. Then, the corresponding eigenfunction correlator $Q_{I}$ of (43) decays exponentially, namely, for any $l\geq 100n_{0}$*

$$Q_{I}\left(0,l\right)\leq 16e^{-\frac{c}{20}l}\equiv 16e^{-c^{\prime }l}.$$

The proof of Lemma 1 is given in Section 4.

**Remark** **5.** *Note that for the case of bounded one-dimensional Schrödinger operator with independent identically distributed potential (aka Anderson model), Lemma 1 combined with the large deviation principle (see [36] and references therein) yields a short proof of the exponential dynamical localisation in expectation* (48)*, the strongest form of Anderson localisation. The first proof of localisation for one-dimensional Anderson model is going back to [37,38]. Since then, the problem has been attracting a considerable attention to this day, see, e.g., Ref. [36] and references therein.*

We conclude this subsection by explaining the choice of spectral interval on which the statements of the above theorems on the area law are valid.

**Remark** **6.** *According to the Definition* (6) *(see also* (41)*) of the Fermi projection, the corresponding spectral interval is $\left[\varepsilon_{-},\varepsilon_{F}\right]$, i.e., between the lower edge of the spectrum $\varepsilon_{-}$ and the given Fermi energy $\varepsilon_{F}$, since it is the set of energies whose states fill the so-called generalized Fermi sphere. This* (45) *and Criterion 2 imply that we have to use the eigenvalue correlator* (43) *and* (44) *with the same spectral interval $\left[\varepsilon_{-},\varepsilon_{F}\right]$.*

*First of all, $\varepsilon_{F}$ has to satisfy* (11) *with I given by* (5)*. Second, $\left[\varepsilon_{-},\varepsilon_{F}\right]$ can include internal spectral gaps, since there the Fermi projection decays exponentially (see item 1 of the text below Definition 1). Third, $\left[\varepsilon_{-},\varepsilon_{F}\right]$ can contain only subsets where the spectrum is pure point, otherwise one cannot guarantee the exponential decay (2.24) of the eigenfunction correlator.*

*This is why there are no restrictions on the value of Fermi energy, hence, on the interval of validity, in Theorems 1 and 2 (ii)–2 (iii). On the other hand, it follows from [39] (see also (Section 18.C in [11])) that the spectrum of the one-dimensional Maryland model is singular continuous on the interval $\left[-\varepsilon_{0},\varepsilon_{0}\right]$, in particular, the ULE property is not valid there (see also Remark 2 about a certain generalisation of the assertion).*

*Likewise, the restriction on the Fermi energy in Theorem 5 is because it follows from [33,34] that their results about pure point spectrum do not apply to a finite set of exceptional points on the spectral axis. Since we use their results, we have to assume that $\varepsilon_{F}$ in Theorem 5 is located to the left of this exceptional set.*

*On the other hand, in all the assertions about the spectrum and the eigenfunction correlator of the operator in question, the corresponding interval can be any one belonging to the pure point spectrum (and, possibly, to the inner spectrum gaps), see formulae* (77)*–*(79) *and Appendix A. In particular, it can be any part of $\sigma_{-}\left(H_{\omega }\right)$ and $\sigma_{+}\left(H_{\omega }\right)$ in Theorem 2 (i) and any part that does not contain exceptional points in Theorem 5 (see also general relations* (45) *and* (46)*).*

### 2.2. Comments and Related Results

We will now comment on various issues related to the above results.

Formulae (7)–(10) for the entanglement entropy of free fermions make natural the following two-step strategy of the large-size asymptotic analysis of the entropy.

The first step is a detailed spectral analysis of the corresponding one-body Hamiltonian (3), in particular, obtaining sufficiently complete information about the matrix (kernel) of the spectral (Fermi) projection (6) or the eigenfunction correlator (43) providing the bounds (38) or (42). Note that this problem has essentially spectral content; moreover, it is one of the main problems of the spectral theory of finite-difference and differential operators.

The second step is an asymptotic analysis of the non-linear and non-smooth functional of the Fermi projection (6) given by the r.h.s. of (7).

Note that these two problems have various amounts of difficulty for different classes of operators and are not always explicitly seen in the work in question. For instance, the Fermi projection of a translation invariant operator (13) can be easily obtained by using the Fourier transformation, and for d=1 is just

(40) $$P\left(\varepsilon_{F}\right)={\left\{P\left(m,n\right)\right\}}_{m,n\in Z},\;\;P\left(m,n\right)=\frac{\sin k_{F}\left(m-n\right)}{\pi (m-n)},$$

where $k_{F}\in R$ is the Fermi momentum determined by the Fermi energy $\varepsilon_{F}$ and the symbol of the operator. However, this simple and explicit expression proved to be of little use in solving the second problem, whose solution requires an involved analysis [40], which becomes quite hard and includes a considerable amount of microlocal analysis in the case of translational invariant pseudodifferential operators in $\ell^{2}\left(Z^{d}\right)$ and $L^{2}\left(Z^{d}\right)$, see [12,13,14,16].

On the other hand, for non-trivial ergodic operators, including the Schrödinger operators $H_{\omega }$ with ergodic random potentials, whose spectrum have a pure point component with exponentially decaying eigenfunctions (Anderson localisation), one expects that if the Fermi energy belongs to the pure point component $\sigma_{pp}\left(H_{\omega }\right)$ of the ω-independent spectrum of $H_{\omega }$, then Fermi projection (6), i.e.,

(41) $$P_{\omega }\left(\varepsilon_{F}\right)=\chi_{\left(\varepsilon_{-},\varepsilon_{F}\right]}\left(H\right)={\left\{P\left(m,n\right)\right\}}_{m,n\in Z^{d}}$$

admits the bound

(42) $$E\left\{|P\left(m,n\right)|\right\}\leq Ce^{-c|m-n|},\;m,n\in Z^{d},$$

where C<∞,c>0, and do not depend on $m,n\in Z^{d}$ but may depend on $\varepsilon_{F}$.

This motivates

**Definition** **1.** *We say that $\lambda_{0}\in R$ is*

*(i) a FPED (Fermi projection exponential decay) point if the Fermi projection $P_{\omega }\left(\varepsilon_{F}\right){|}_{\varepsilon_{F}=\lambda_{0}}$ of* (41) *satisfies* (42)*,*

*(ii) an AL (Area Law) point if the expectation $E\{S_{\Lambda }\left(\varepsilon_{F}\right){|}_{\varepsilon_{F}=\lambda_{0}}\}$ of the entanglement entropy* (7)*–*(10) *obeys the Area Law* (1)*.*

Here are two types of FPED-points.

(1) Any point of an internal gap of the spectrum σ(H) of the one-body Hamiltonian *H* of (14) and (15). Indeed, it follows from the Combes–Thomas estimate (Section 10.3 in [10]) and (Appendix B in [35]) that the Green function, ${\left(H-z\right)}^{-1}\left(m,n\right),\;m,n\in Z^{d}$, of a (non necessarily ergodic) operator (14) decays exponentially if z∈C∖σ(H). Combining this with the contour integration trick (see, e.g, (Section 13 in [10])) we obtain the exponential decay of the Fermi projection $P(\varepsilon_{F})$ if the Fermi energy $\varepsilon_{F}$ falls in a gap.

(2) Any point of the pure point spectrum with the exponentially decaying eigenfunctions of $H_{\omega }$ with independent identically distributed (monotone) random potentials, see (14), (15) and (17), i.e., for the case of so-called “strong” Anderson localisation, mentioned above. For this case, the proof of (42) is among the top results of the field, see, e.g., (Section 13 in [10]) and [41] for any d≥1, and (Section 12 in [10]) for d=1, where the whole spectrum is strongly localised.

Note that the same (FPED) property holds for more general classes of random operators, the so-called Wegner orbital model and Schrödinger operators with non-monotone independent identically distributed random potentials, see [42,43]. Thus, Criterion 1 below yields the validity of the Area Law for the expectation of the entanglement entropy of these models.

The bound (42) provides the solution of the first problem. Moreover, it proves to be the main ingredient in the solution of the second problem, hence, in obtaining the Area Law for the Schrödinger operators with random potentials, see [17].

Denote $\sigma_{FPDE}\left(H\right)$ and $\sigma_{AL}\left(H\right)$ the sets of the FPDE- and AL-points of our one-body Hamiltonian. Then we can formulate the main result of [17] (the solution of the second problem for the random Schrödinger operator) as the proof of the inclusion

$$\sigma_{FPED}\left(H\right)\subseteq \sigma_{AL}\left(H\right).$$

However, as the analysis of the proof of the Area Law in (Results 2–3 in [17]) shows, it is applicable to any ergodic finite-difference operator for which the estimate (42) is valid.

The goal of this work is to use the same approach, i.e., the analysis of $\sigma_{FPED}$, to prove the Area Law for certain dynamically generated (including some quasi-periodic) potentials in (14) and (15).

It is convenient to formulate the above as follows.

**Criterion** **1.** *Let $S_{\Lambda }\left(\varepsilon_{F}\right)$ be the entanglement entropy* (7)*–*(10) *of free lattice fermions whose one-body Hamiltonian $H_{\omega }$ is a self-adjoint ergodic operator (12) in $\ell^{2}\left(Z^{d}\right)$, and let $P_{\omega }$ be its Fermi projection (6). Assume that the Fermi energy $\varepsilon_{F}$ belongs to $\sigma_{FPED}$(H), i.e.,* (42) *holds.*

*Then, for $\Lambda =\Lambda_{M}={\left[-M,M\right]}^{d}\subset Z^{d},\;L=2M+1$, the expectation of the corresponding entanglement entropy $S_{\Lambda }\left(\varepsilon_{F}\right)$ of* (7) *obeys the Area Law:*

$$0<\lim_{L\to \infty }L^{-(d-1)}E\;\left\{S_{\Lambda_{M}}\left(\varepsilon_{F}\right)\right\}:=s_{d}\left(\varepsilon_{F}\right)<\infty ,$$

*see ref. [17] for an explicit formula for $s_{d}\left(\varepsilon_{F}\right)$.*

Our strategy is essentially determined by Criterion 1, i.e., it consists of proving the exponential bound (42) for several classes of non-random ergodic operators.

It turns out that in a number of cases under consideration, it is convenient to use a spectral characteristic somewhat more general but simpler than the Fermi projection and known as the *eigenfunction correlator*, introduced by Aizenman [44], see also (Section 1.4, and Chapter 7 in [10]) and Definition 2 below. The characteristic proves to be important in a number of problems of spectral analysis of ergodic operators and its applications, see, e.g., Refs. [10,11,41] and references therein.

**Definition** **2.** *Let H be a self-adjoint operator on $\ell^{2}\left(Z^{d}\right)$. Then, the eigenfunction correlator for a Borel set I⊂R and any $m,n\in Z^{d}$ is*

(43) $$Q_{I}\left(m,n\right):=\underset{\phi }{\sup}\left\{\right|\langle \delta_{m},\phi \left(H\right)\chi_{I}\left(H\right)\delta_{n}\rangle {|:\;\phi \in C\left(R\right),\;\;∥\phi ∥}_{\infty }\leq 1\}.$$

*where C(R) is the space of continuous real-valued functions, ${\left\{\delta_{n}\right\}}_{n\in Z^{d}}$ is the canonical basis of $\ell^{2}\left(Z^{d}\right)$, and $\chi_{I}$ is the indicator of I.*

If the spectrum of *H* in *I* is pure point, and ${\left\{\lambda_{l}\right\}}_{l}$ and ${\left\{\psi_{l}\right\}}_{l}$ are the eigenvalues (counted with their multiplicity) and normalized eigenfunctions, respectively, then

(44) $$Q_{I}\left(m,n\right)=\sum_{\lambda_{l}\in I}|\psi_{l}\left(m\right)|\;|\psi_{l}\left(n\right)|.$$

This explains the name of $Q_{I}$.

Next, we have, from the spectral theorem, (41) and (44) with $I=[\varepsilon_{-},\varepsilon_{F})$

(45) $$\left|P\left(m,n\right)\right|\leq Q_{\left[\epsilon_{-},\varepsilon_{F}\right)}\left(m,n\right).$$

We also have

(46) $$\underset{t\in R}{\sup}\left\{\right|\langle \delta_{m},e^{-itH}\chi_{I}\left(H\right)\delta_{n}\rangle |\leq Q_{I}\left(m,n\right),$$

where the l.h.s. is another spectral characteristic, important in applications, in particular, in determining the *exponential dynamical localisation in expectation* (see (48)), one of the strongest forms of the Anderson localisation.

The above bounds and Criterion 1 imply the following workable criterion for the validity of the Area Law and the exponential dynamical localisation in expectation.

**Criterion** **2.** *Let $H_{\omega }$ be a self-adjoint ergodic operator (12) in $\ell^{2}\left(Z^{d}\right)$ and $Q_{I}\left(m,n\right)$ be its eigenfunction correlator (43) with a Borel set I∈R. Then, the bound*

(47) $$E\{|Q_{I}\left(m,n\right)\left|\right\}\leq Ce^{-c|m-n|},\;m,n\in Z^{d},\;$$

*where C<∞ and c>0 do not depend on m,n (but may depend on I), implies the following:*

*(i) Exponential dynamical localisation in expectation of $H_{\omega }$ on I:*

(48) $$E\left\{\underset{t\in R}{\sup}|\langle \delta_{m},e^{-itH_{\omega }}\chi_{I}\left(H_{\omega }\right)\delta_{n}|\right\}\leq Ce^{-c|m-n|}.$$

*(ii) Area Law (1) for the expectation $E\{S_{\Lambda }\left(\varepsilon_{F}\right)\}$ of the entanglement entropy* (7)*–*(10) *of free lattice fermions having $H_{\omega }$ as their one-body Hamiltonian if $I=(\varepsilon_{-},\varepsilon_{0}]$ is the bottom of the spectrum of $H_{\omega }$ and the Fermi energy $\varepsilon_{F}<\varepsilon_{0}$.*

We will consider first the finite-difference operators (14) with quasi-periodic potentials for which exponential bounds for the Fermi projection (41) and/or the eigenfunction correlator (43) are essentially known, although sometimes in the form that has to be made suitable for our purposes. These results are presented largely for the sake of completeness and also to demonstrate yet another application of the spectral theory of ergodic operators, this time to the quantum information science.

To explain our main new result, note first that quasi-periodic functions follow the orbits of the irrational winding (total shifts) of the torus $T^{d}$, a quite simple dynamical system. A considerably more complex and rich class of dynamical systems with the same phase space $T^{d}$ consists of the so-called subshifts of finite type [23,24,45]. The spectral analysis of these operators has been recently developed in [33,34]. A number of important and highly non-trivial facts, including the existence of Anderson (spectral) localisation, was established in these papers. However, the crucial for our purposes exponential decay (47) of the eigenfunction correlator and/or Fermi projection (42) is lacking.

We will prove this property, hence, by Criterion 2, obtaining the Area Law, as well as the exponential dynamical localisation in expectation, which we believe is of independent interest.

The exponential bounds (42) and (48) are well known in the spectral theory of random ergodic operators, first of all the Schrödinger operators whose potential is a collection of independent identically distributed random variables [10,41,42]. There are several proofs of the bounds in this case, a quite streamlined and efficient one is based on the analysis of the fractional moments of the resolvent of the operator.

The proofs of (42) and (48) for the quasi-periodic operators are based on the positivity of the Lyapunov exponent (see, e.g., (87)) of the corresponding finite-difference equation and on the related exponential decay of the eigenfunctions of $H_{\omega }$. We will use this approach for Theorems 1 and 2 in Section 3.

The proof of Theorems 4 and 5 for the Schrödinger operators whose potential is generated by a subshift of finite type is using as an input the uniform positivity of the Lyapunov exponent and the uniform large deviation-type estimate from [33,34]. In addition, we use special techniques of the theory of dynamical systems involving the Markov partitions and related Markov chains [23,24].

## 3. Finite-Difference Operators with Quasi-Periodic Potential

In this section, we present the proofs of Theorems 1 and 2 on the Area Law for several classes of non-random ergodic operators. We begin with the proof of Theorem 3, which is important as itself and crucial in the proofs of Theorems 1 (i) and 2 (i).

To the best of our knowledge, the only class of ergodic operators for which the Area Law is rigorously established (using Criterion 1) is that consisting of the Schrödinger operators with independent identically distributed random potentials (also known as the Anderson model) [17].

Here, we show that the class of such operators is considerably larger. In particular, it includes several families of quasi-periodic (and limit-periodic), i.e., deterministic, rather than random, operators. These are operators given by (4), whose ergodic potential (17) is generated by an irrational shift

T(ω)=ω+αmod1,ω∈T,α∈R∖Q,n∈Z,

that is, Ω=T equipped with the Borel σ-algebra and P being the normalized Lebesgue measure.

The two archetypal examples of such one-dimensional Schrödinger operators are the Maryland model, whose potential is (28), and the almost Mathieu operator with potential (32).

We start with the proof of Theorem 3, which we consider to be of independent interest.

### 3.1. Uniformly Localised Eigenfunctions for the d-Dimensional, d≥1, Maryland Model

**Proof of Theorem 3** **(i).** According to (Sections 18.A–18.B in [11]), the spectrum of the *d*-dimensional Maryland model (see (14)–(15) and (18)–(19)) occupies the whole R, is pure point, of multiplicity 1, and is described as follows. There exist a 1-periodic and monotone on the period function $\lambda_{0}:T\to R$ and

(49) $$\psi_{0}:R\times Z^{d}\to C,\;\sum_{n\in Z^{d}}{\left|\psi_{0}\left(\lambda ,n\right)\right|}^{2}=1,\;\forall \lambda \in R,$$

such that the eigenvalues ${\left\{\lambda_{l}\left(\omega \right)\right\}}_{l\in Z^{d}}$ are

(50) $$\lambda_{l}\left(\omega \right)=\lambda_{0}(\omega +\langle \alpha ,l\rangle ),\;\lambda_{l_{1}}\left(\omega \right)\neq \lambda_{l_{2}}\left(\omega \right)⟺l_{1}\neq l_{2},$$

and the corresponding eigenfunctions ${\left\{\psi_{l}\left(\omega \right)\right\}}_{l\in Z^{d}}$, $\psi_{l}\left(\omega \right)={\left\{\psi_{l}\left(\omega ,n\right)\right\}}_{n\in Z^{d}}$ are

(51) $$\psi_{l}\left(\omega ,n\right)=\psi_{0}\left(\lambda_{l}\left(\omega \right),n-l\right).$$

Note first that the function $\lambda_{0}$ of (50) is the functional inverse of the Integrated Density of States N(λ) of $H_{\omega }$ (see (Sections 4.B–4.C in [11]) and (Sections 3.3–3.4 in [10]) for its definition and properties). We have for the Maryland model

(52) $$N\left(\lambda \right)=\int_{-\infty }^{\lambda }n\left(\lambda \right)d\lambda ,$$

and,

(53) $$\lambda_{0}\circ N=1,$$

and

(54) $$n\left(\lambda \right)=\frac{g}{{\left(2\pi i\right)}^{d}\pi }\int_{T^{d}}{\left|w\left(\eta \right)-\lambda +ig\right|}^{-2}\prod_{j=1}^{d}\frac{d\eta_{j}}{\eta_{j}},$$

is the Density of States of $H_{\omega }$, and $w:T^{d}\to R$ is given by the *d*-dimensional Fourier series with coefficients of (15)

(55) $$\begin{array}{cc} \; & \;w\left(\eta \right)=\sum_{n\in Z^{d}}W\left(n\right)\;\eta_{1}^{n_{1}}\cdots \eta_{d}^{n_{d}},\\ \eta & =\left(\eta_{1},\ldots ,\eta_{d}\right)\in T^{d},\;\;n=\left(n_{1},\ldots ,n_{d}\right)\in Z^{d}. \end{array}$$

Next, the function $\psi_{0}$ of (49) is (see (Section 18.B in [11]))

(56) $$\psi_{0}\left(\lambda ,n\right)=\frac{1}{{\left(2\pi i\right)}^{d}{\left(\pi n\left(\lambda \right)/g\right)}^{1/2}}\int_{T^{d}}\frac{e^{2\pi it(\lambda ,\eta )}}{\left(w(\eta )-\lambda -ig\right)}\prod_{j=1}^{d}\eta^{n_{j}-1}d\eta_{j},$$

where

(57) $$t\left(\lambda ,\eta \right)=\sum_{m\in Z^{d}}t_{m}\left(\lambda \right)\prod_{j=1}^{d}\eta_{j}^{m_{j}},\;t_{m}\left(\lambda \right)=\frac{l_{m}\left(\lambda \right)}{\left(1-e^{i\langle \alpha ,m\rangle }\right)}$$

with

(58) $$l_{m}\left(\lambda \right)={\left(2\pi i\right)}^{-d}\int_{T^{d}}\log c\left(\lambda ,\eta \right)\prod_{j=1}^{d}\eta^{m_{j}-1}d\eta_{j},$$

and

(59) $$c\left(\lambda ,\eta \right)=-\frac{w(\eta )-\lambda +ig}{w(\eta )-\lambda -ig}.$$

Thus, the c(λ,η) of (59) is the input of the above analytic procedure given by formulae from (56) to (58) that leads to the eigenfunctions (51).

Our goal is to prove the bound (33). This, combined with Remark 2 and Criterion 2, will allow us to establish the validity of the Area Law for the Maryland model.

We begin with applying Lemma 2 (i) to *w* of (55) whose Fourier coefficients satisfy (15), and we find that *w* admits the analytic continuation to $T_{\rho_{1}}^{d},\;0<\rho_{1}<\rho$, where ρ>0 is given in (15). Moreover, since the “initial” *w* of (55) is real-valued ($w:T^{d}\to R$), we have for its analytic continuation

(60) limr→0Imw(erη)=0,|η|=1.

We conclude that there exists $0<\rho_{2}<\rho_{1}$ such that for any $\left|r\right|\leq \rho_{2}$

(61) |w(erη)−λ±ig|≥|g±Imw(erη)|≥|g|−|Imw(erη)|≥g/2,

where the last inequality follows from (60). We have then from (59) that |logc(λ,η)|≤|log|c(λ,η)||+2π, and for any $\left|r\right|\leq \rho_{2}$

$$\begin{array}{ccc} \left|\log|c(\lambda ,e^{r}\eta )|\right| & = & |\log|1+2ig{\left(w\left(e^{r}\eta \right)-\lambda -ig\right)}^{-1}\left|\right|\\ & \leq & 2|g|\;|w\left(e^{r}\eta \right){-\lambda -ig|}^{-1}\leq 4, \end{array}$$

where in obtaining the last bound of the r.h.s. above, we used (61). Combining the two last bounds, we find for any $\left|r\right|\leq \rho_{2}$

$$|\log c(\lambda ,e^{r}\eta )|\leq C,$$

where *C* is a constant. Now combining (58) and Lemma 2 (ii), we obtain

$$|l_{m}\left(\lambda \right)|\leq Le^{-\rho_{2}\left|m\right|},$$

where L<∞ and $\rho_{2}>0$ do not depend on λ and m.

This and the Diophantine condition (19) imply for the Fourier coefficients $t_{m}\left(\lambda \right)$ of (57)

(62) $$|t_{m}\left(\lambda \right)|\leq Le^{-\rho_{2}^{\prime }\left|m\right|},\;0<\rho_{2}^{\prime }<\rho_{2}.$$

Then, Lemma 2 (ii) implies that t(λ,·) of (57) admits the analytic continuation into $T_{\rho_{3}}^{d}$ with some $0<\rho_{3}<\rho_{2}^{\prime }$ and is bounded there for any $\left|r\right|\leq \rho_{3}$, i.e.,

(63) $$|t\left(\lambda ,e^{r}\eta \right)|\leq T<\infty ,\;|r|\leq \rho_{3},$$

where *T* and $\rho_{3}$ are λ-independent according to (73).

Next, (61) and the analyticity of *w* in $T_{\rho_{1}}^{d}$ imply that

$$h\left(\lambda ,z\right)=\frac{e^{t(\lambda ,z)}}{{\left(\pi n\left(\lambda \right)/g\right)}^{1/2}\left(w\left(z\right)-\lambda -ig\right)}$$

is analytic in z in $T_{\rho_{3}}^{d}$ for any λ∈R. Since the r.h.s. of (56) is the Fourier coefficient of h(λ,·), (74) yields the following bound for the r.h.s. of (56)

(64) $$\Psi e^{-\rho_{3}\left|n\right|},\;n\in Z^{d},\;\Psi =\max_{\lambda \in R,\;|r|\leq \rho_{3},\;\left|\eta \right|=1}\left|h\left(\lambda ,e^{r}\eta \right)\right|.$$

It follows from (54) that

1. ${\left(n\left(\lambda \right)\right)}^{-1/2}\geq {\left(\pi g\right)}^{1/2},$ and ${\left(n\left(\lambda \right)\right)}^{1/2}={\left|\lambda \right|}^{-1}\left(1+o\left(1\right)\right),\;\left|\lambda \right|\to \infty$, by (54);
2. $|w(z)-\lambda -ig|,\;|z|\in \left[e^{-\rho_{3}},e^{\rho_{3}}\right]$ is bounded from below by (61) and is |λ|(1+o(1)),|λ|→∞;
3. $e^{t(\lambda ,z)}\leq e^{T}$ by (63).

This and (64) imply that Ψ is independent of λ∈R and $n\in Z^{d}$.

Thus, we obtain

(65) $$|\psi_{0}\left(\lambda ,n\right)|\leq Ce^{-c|n|},\;n\in Z^{d},$$

with Ψ of (64) as *C* and $\rho_{3}$ of (63) as *c*. Now (50)–(51) imply

(66) $$|\psi_{l}\left(\omega ,n\right)|\leq Ce^{-c|n-l|},$$

where C<∞ and c>0 are independent of ω. □

**Proof of Theorem 3** **(ii).** According to (Section 18.C in [11,39]), the pure point spectrum consists of two semi-infinite components (29).

Since, in this case, operator *W* in (14) is the one-dimensional discrete Laplacian, we set $W_{0}=0,\;W_{\pm 1}=1$ in (15). Hence, *w* of (55) is

(67) $$w\left(\eta \right)=\eta +\eta^{-1}.$$

Thus, *w* is analytic everywhere except zero and infinity, i.e., in this case, the analog of $\rho_{1}$ of the proof above is infinity.

Next, the integral in the r.h.s. of Formula (58) for d=1 and *w* of (67) can be calculated, yielding (cf. (58))

$$l_{m}=\frac{2i}{\left|m\right|}e^{-\gamma |m|}\sin\left|m\right|\varphi .$$

Here, $e^{-\gamma -i\varphi }=:\eta_{0}$ is the root of equation $\eta +\eta^{-1}=\lambda +ig$, such that $|\eta_{0}|<1$ and γ:=γ(λ,g) is the Lyapunov exponent of the corresponding finite-difference equation

(68) $$u_{n+1}+u_{n-1}+g\tan(\alpha n+\omega )u_{n}=\lambda u_{n}.$$

We have

(69) sinhγ(λ,g)=s>0,

where s>0 such that

(70) $$s^{4}+\left(4-\lambda^{2}-g^{2}\right)s^{2}-g^{2}=0,$$

implying that γ(λ,g) is an even and convex function of λ∈R and

(71) γ(λ,g)≥γ(0,g)=arcsinh(|g|/2)>0,g≠0.

Denote $\lambda_{0}\geq 0$ the root of equation γ(λ,g)=β(α), with β(α) defined in (27), and fix $\varepsilon_{0}>\lambda_{0}$. Then, setting

$$\rho^{\prime }:=\gamma \left(\varepsilon_{0},g\right)-\beta \left(\alpha \right)>0,$$

we obtain from (57) and (58) for the Fourier coefficients $t_{m}\left(\lambda \right)$ of function t(η,·) of (57) for d=1

$$|t_{m}\left(\lambda \right)|\leq Ce^{-\rho^{\prime }\left|m\right|},\;\left|\lambda \right|\geq \varepsilon_{0}.$$

This bound is an analog of (62). Hence, repeating the argument of the previous proof starting from (57), we obtain

(72) $$|\psi_{l}\left(\omega ,m\right)|\leq Ce^{-c|m-l|},\;m\in Z,\;\left|\lambda \right|\geq \varepsilon_{0},$$

with ω-independent parameters C<∞,c>0, and $\varepsilon_{0}\geq 0$. □

**Lemma** **2.** *Let $f:T^{d}\to C$ be a periodic function and ${\left\{F_{n}\right\}}_{n\in Z^{d}}$ be its Fourier coefficients. We have*

*(i) If $|F_{n}|\leq Fe^{-\rho_{1}\left|n\right|},\;F<\infty ,\;\rho_{1}>0,\;n\in Z^{d},$ then f admits the analytic continuation into*

$$T_{\rho_{1}}^{d}=\prod_{j=1}^{d}T_{\rho_{1}},\;T_{\rho_{1}}=\{z\in C:e^{-\rho_{1}}\leq \left|z\right|\leq e^{\rho_{1}}\},$$

*where for any $0<\rho_{2}<\rho_{1}$ and $a=\rho_{1}-\rho_{2}>0$, it has the bound*

(73) $$\left|f\left(z\right)\right|\leq F{\left(\frac{e^{a}+1}{e^{a}-1}\right)}^{d},\;for\;any\;z\in T_{\rho_{2}}^{d}.$$

*(ii) If f admits the analytic continuation to $T_{\rho_{1}}^{d},\;\rho_{1}>0,$ then*

(74) $$\begin{array}{ccc} |F_{n}| & \leq & Fe^{-\rho_{1}\left|n\right|},\;F<\infty ,\;n\in Z^{d},\\ F & = & \max_{\left|r\right|\leq \rho_{1},\;\left|\eta \right|=1}\left|f\left(e^{r}\eta \right)\right|. \end{array}$$

**Proof.**  (i). Write the first line of (3.13) as $|F_{n}|\leq F\prod_{j=1}^{d}e^{-\rho_{1}\left|n_{j}\right|}$. Since the multidimensional Fourier series is in our notation

$$f\left(\eta \right)=\sum_{n\in Z^{d}}F_{n}\eta^{n},\;\eta^{n}=\eta_{1}^{n_{1}}\cdots \eta_{d}^{n_{d}},\;\left|\eta_{j}\right|=1,\ldots ,d,\;$$

we have for $z^{n}=z_{1}^{n_{1}}\cdots z_{d}^{n_{d}}=e^{r}\eta^{n}\;\left|r\right|<\rho_{1}$:

$$\left|f\left(z\right)\right|\leq F\prod_{j=1}^{d}\sum_{n_{j}\in Z}e^{-\rho_{1}\left|n_{j}\right|}{\left|z_{j}\right|}^{n_{j}}.$$

The sum in the r.h.s. with $\left|r\right|\leq \rho_{2}<\rho_{1}$ is bounded by

$$\sum_{n_{j}\in Z}e^{-\rho_{1}\left|n_{j}\right|}e^{\rho_{2}n_{j}}\leq 1+2\sum_{n_{j}=1}^{\infty }e^{-a}=\left(e^{a}+1\right)/\left(e^{a}-1\right).$$

Plugging this bound into the r.h.s. of the previous formula, we obtain assertion (i).

(ii) We have in our notation for the Fourier coefficients of *f*

(75) $$F_{n}={\left(2\pi i\right)}^{-d}\int_{|\eta |=1}f\left(\eta \right)\prod_{j=1}^{d}\eta_{j}^{n_{j}-1}d\eta_{j}.$$

Consider first the case where d=1 and

$$F_{n}={\left(2\pi i\right)}^{-1}\int_{|\eta |=1}f\left(\eta \right)\eta^{n-1}d\eta .$$

Assume that n>0. Since *f* is analytic in $T_{\rho_{1}}$, we can deform the integration contour over the unit circle to that over $\left|z\right|=e^{-\rho_{1}}$ and obtain

$$|F_{n}{|\leq \left(2\pi \right)}^{-1}Fe^{-\rho_{1}n}\int_{|\eta |=1}\left|d\eta \right|=Fe^{-n\rho_{1}}.$$

If n<0, then we deform the contour to $\left|z\right|=e^{+\rho_{1}}$ and repeat the above argument.

This yields the one-dimensional version of bound in assertion (ii). To obtain its multidimensional version, we apply the above argument to every integration in (75). □

### 3.2. Proof of Theorem 1

**Proof of Theorem 1** **(i).** Spectral theorem for the Fermi projection

(76) $$P\left(\omega ,m,n\right)=\sum_{\lambda_{l}\left(\omega \right)\leq \varepsilon_{F}}\psi_{l}\left(\omega ,m\right)\bar{\psi_{l}\left(\omega ,n\right)}$$

combined with (66) imply

(77) $$\begin{array}{cc} \; & \left|P(\omega ,m,n)\right|\leq Q_{\left[\varepsilon_{-},\varepsilon_{F}\right)}\left(\omega ,m,n\right)=\sum_{l:\lambda_{l}\left(\omega \right)\leq \varepsilon_{F}}\left|\psi_{l}\left(\omega ,m\right)\psi_{l}\left(\omega ,n\right)\right|\\ \; & \;\leq C^{2}\sum_{l:\lambda_{l}\left(\omega \right)\leq \varepsilon_{F}}e^{-c|m-l|-c|n-l|}. \end{array}$$

Now, the triangle inequality |m−l|+|n−l|≥|m−n| yields the following upper bound for the r.h.s. (77):

(78) $$C^{2}e^{-c|m-n|/2}\sum_{l\in Z^{d}}e^{-c|m-l|/2}=\tilde{C}e^{-\tilde{c}\left|m-n\right|},$$

where

(79) $$\tilde{C}=C^{2}\sum_{m\in Z^{d}}e^{-c|m|/2}<\infty .$$

Combining (76)–(79), we obtain an analog of (42) and (47) with some ω-independent $c=\tilde{c},\;C=\tilde{C}$ for $P_{\omega }\left(\varepsilon_{F}\right)$ and $Q_{I}$, but not for their expectations. Since, however, $c=\tilde{c}$ and $C=\tilde{C}$ are ω-independent, the same bounds hold for the corresponding expectations in Criterion 1 and Criterion 2, which, in turn, imply the validity of the Area Law for the Maryland model. □

Here is one more example where the localisation centres are also explicit and non-random as in the Maryland model.

**Proof of Theorem 1** **(ii).** Kachkovskiy, Parnovski, and Shterenbereg consider in [46] a class of *d*-dimensional Schrödinger operators (4) with potentials (20) described in the assertion, i.e., with ξ-Hölder 1-periodic monotone sample functions (21) and weakly Diophantine frequencies (22).

An archetypal example in this class of operators is the multidimensional Maryland model from Theorem 1 (i). However, unlike the above class of operators, the Maryland model is explicitly solvable for any non-zero coupling constants *g* and for the Diophantine frequencies (19).

The main (perturbative) result of [46] states the following:

**Proposition** **1** (Theorem 1.1 in [46])**.** *Let ξ≥1,ρ,μ>0,0<δ<1. There exists $g_{0}=g_{0}\left(d,\rho ,\mu ,\xi ,\delta \right)>0$ such that for every $g>g_{0},\;\alpha \in \Omega_{\rho ,\mu }$, and ξ-Hölder (1-periodic) monotone function v:R→[−∞,+∞), one can find a* 1*-periodic function λ:R→[−∞,+∞), strictly increasing on [0,1), and a 1-periodic measurable function $\psi :R\to \ell^{2}\left(Z^{d}\right)$ such that*

(80) $$H_{\omega }\psi \left(\omega \right)=\lambda \left(\omega \right)\psi \left(\omega \right),\;for\;all\;\;\omega \in R,$$

*and*

$$\begin{array}{cc} \; & {∥\psi \left(\omega \right)∥}_{\ell^{2}\left(Z^{d}\right)}=1,\;\left|\psi \left(\omega ,0\right)-1\right|<g^{-(1-\delta )}\\ \; & \;|\psi \left(\omega ,n\right)|\leq g^{-(1-\delta )|n|}\;\;for\;\;\left|n\right|\neq 0, \end{array}$$

*where $\psi \left(\omega ,n\right)=\langle \psi \left(\omega \right),\delta_{n}\rangle$ denotes the components of the vector-valued function ψ, and ${\left\{\delta_{n}\right\}}_{n\in Z^{d}}$ denotes the canonical basis of $\ell^{2}\left(Z^{d}\right)$.*

It follows then from the proposition that

- the spectrum of $H_{\omega }$ occupies the whole R, is pure point and of multiplicity 1, its eigenvalues ${\left\{\lambda_{l}\left(\omega \right)\right\}}_{l\in Z^{d}}$ and the corresponding eigenfunctions ${\left\{\psi_{l}\left(\omega \right)\right\}}_{l\in Z^{d}}$, $\psi_{l}\left(\omega \right)={\left\{\psi_{l}\left(\omega ,n\right)\right\}}_{n\in Z^{d}}$ are
  (81) $$\lambda_{l}\left(\omega \right)=\lambda \left(\omega +\langle \alpha ,l\rangle \right),\;\psi_{l}\left(\omega ,n\right)=\psi \left(\omega +\langle \alpha ,l\rangle ,n-l\right),$$
- the eigenfunctions admit the bound $|\psi_{l}\left(\omega ,n\right)|\leq Ce^{-c|n-l|}$ with the ω-independent C<∞ and c>0, i.e., we have the ULE property (36).

Thus, the description of the spectrum for this class of operators coincides, at least for large *g*, with that of (49)–(51) for the Maryland model and admits the exponential (ULE) bound (36) analogous to (33). Thus, repeating literally the proof of Theorem 1 (i), we arrive to the same conclusion on the validity of the Area Law (and the exponential dynamical localisation in expectation) on the whole spectrum of the operators in question. □

**Remark** **7.** *The relations* (80) *and* (81) *are known as the covariant spectral representation and can also be easily checked in the Maryland model. For their general discussion, see [47].*

**Proof of Theorem 1** **(iii).** Ge, You, and Zhou consider in [48] the quasi-periodic operators (14) and (15) with the multidimensional analog (23) of the almost Mathieu potential (32). They establish the following.

**Proposition** **2** (Theorem 1.2 in [48])**.** *For $\alpha \in DC_{d}$ of* (24)*, there exists $g_{0}\left(\alpha ,d\right)>0$ such that if $g>g_{0}$, then the operator* (14) *and* (15) *with potential* (32) *exhibits exponential dynamical localisation in expectation (48).*

However, as follows from the analysis of the proof, the only property of the evolution operator $e^{-itH}$ that is used in [48] is the bound $\left|\right|e^{-itH}\left|\right|\leq 1$ that follows from the elementary bound $|e^{-itx}|\leq 1$ of the exponential function. Thus, replacing this function by a bounded continuous ϕ of Definition 2, we obtain the bound (47) instead of (48), and then Criterion 2 implies the assertion (iii) of Theorem. □

**Proof of Theorem 1** **(iv).** We begin with two definitions (see [25,26,27,28] for details).

**Definition** **3.** *(i)* Ω *is a Cantor group if it is an infinite, totally disconnected, compact Abelian topological group with no isolated points. In that case,* Ω *has a unique translation invariant probability measure, Haar measure. One fixes a metric on* Ω *that is compatible with Haar measure.*

*(ii) Consider a Cantor group* Ω *and a $Z^{d}$ action by translations, ${\left\{T^{n}\right\}}_{n\in Z^{d}}$. Namely, there are $\omega_{1},\ldots ,\omega_{d}\in \Omega$ such that for every ω∈Ω,*

$$T^{n}\omega =\omega +\sum_{j=1}^{d}n_{j}\omega_{j},\;n=\left(n_{1},\ldots ,n_{d}\right)\in Z^{d},$$

*where + denotes the group operation. The action is called minimal if all orbits are dense, namely, if $\bar{\left\{T^{n}\omega :n\in Z^{d}\right\}}=\Omega$ for every ω∈Ω.*

Damanik and Gan consider in [25] a class of *d*-dimensional Schrödinger operators (4) with potential defined by $V_{\omega }\left(n\right)=f\left(T^{n}\omega \right)$ as in (25), where ω∈Ω is a Cantor group that admits a minimal $Z^{d}$ action *T* by translations, and f∈C(Ω,R). The main result of [25] states the following:

**Proposition** **3** (Theorem 1.3 in [25])**.** *There exists a Cantor group* Ω *that admits a minimal $Z^{d}$ action T by translation and an f∈C(Ω,R) such that for every ω∈Ω, the Schrödinger operator* (4) *with potential $f(T^{n}\omega )$ of* (25) *has ULE* (37) *with ω-independent constants.*

We conclude that the description of the spectrum for this class of operators coincides with that of (49)–(51) for the Maryland model and admits the exponential (ULE) bound (36) analogous to (33). Thus, repeating literally the proof of Theorem 1 (i), we arrive to the same conclusion on the validity of the Area Law (and the exponential dynamical localisation in expectation) on the whole spectrum of the operators in question. □

### 3.3. Proof of Theorem 2

We will now pass to the proof of Theorem 2, dealing with one-dimensional almost periodic operators.

**Proof of Theorem 2** **(i).** The estimate (72) allows us to apply again formulae (76)–(79) to obtain (42), and then Criterion 1 implies the assertion of Theorem 2 (i). □

**Proof of Theorem 2** **(ii).** The Lipschitz monotone potentials (30) and (31) are similar to the potential (28) of Maryland model; however, they are bounded. That is, the periodic sample function *v* in (30) is strictly increasing on [0,1) but has the jump discontinuities at integer points. So the potential graph has a sawtooth shape.

Jitomirskaya and Kachkovskiy consider in [49] the one-dimensional discrete Schrödinger operators with this class of potentials and establish the following:

**Proposition** **4** (Corollary 3 in [32])**.** *Suppose α is Diophantine, namely, there exist C<∞,τ>0 such that ${∥n\alpha ∥>C|n|}^{-\tau },\;n\in N$, where ∥x∥=min({x},{1−x}). Let $\epsilon \left(\alpha \right)={lim inf}_{k}q_{k-1}/q_{k+1}$, where $\left\{q_{k}\right\}$ are the denominators of the continued fraction approximants of α (note that ϵ(α)≤1/2 for any α∈R∖Q). Suppose that $g>2e/\left(\left(1-\epsilon \left(\alpha \right)\right)a_{-}\right)$.*

*Then, there exist C<∞,c>0 such that for any orthonormal eigenfunction $\psi_{l}\left(\omega \right)$ there exists $n_{l}\left(\omega \right)$ such that we have for $u_{l}\left(\omega \right)=\psi_{l}\left(\omega \right)/\psi_{l}\left(\omega ,0\right)$*

(82) $$|u_{l}\left(\omega ,n\right)|<Ce^{-c|n-n_{l}\left(\omega \right)|},$$

*where C<∞ and c>0 are ω-independent.*

This bound is similar to the one of [50] but is valid for all ω. However, unlike (72), or, more generally, (66), the bound (82) cannot be used immediately to prove the exponential decay of the Fermi projector and/or the eigenfunction correlator, as was possible with the estimate (33) and (66), see Formulae (33) and (79). The reason is that the functions ${\left\{u_{l}\right\}}_{l}$, although orthogonal, are not orthonormalized. Therefore, an additional argument must be used to pass from ${\left\{u_{l}\right\}}_{l}$ to ${\left\{\psi_{l}\right\}}_{l}$ in the above bound. Such an elegant argument was proposed in [50]. Thus, by using this argument and (82), it is possible to prove the exponential dynamical localisation in expectation as well as the exponential decay of the Fermi projection, the eigenfunction correlator, and the assertion of Theorem 2 (ii). □

**Proof of Theorem 2** **(iii).** In the work [50], the exponential dynamical localisation in expectation (48) was proven under the conditions of Theorem 2 (iii). However, just like in [48] (see the proof of Theorem 1 (iii)), the only property of the operator $e^{-itH}$ that was used in [50] was the bound $\left|\right|e^{-itH}\left|\right|\leq 1$. Therefore, we repeat our argument from the proof of Theorem 1 (iii), allowing for the replacement of $e^{-itH}$ by any ϕ(H) of Definition 2. We obtain a more general bound (47) instead of (48), and then Criterion 2 implies the assertion of Theorem 2 (iii). □

## 4. Proof of General Criterion for “Strong” Dynamical Localisation

Here, we prove Lemma 1. The lemma is one of possible rigorous implementations of the heuristics of quantum mechanics, according to which tunneling between two potential wells is suppressed if the wells are sufficiently distant from each other and sufficiently different so that the distances between their eigenvalues are not too small. The use of resolvent identities in the proof of various implementations was initiated in the seminal paper [35].

Let *G* be the resolvent operator of *H* and $G_{\left[m,p\right]}$ be the resolvent operator of the restricted operator $H^{\left[m,p\right]}$. Let *n* be such that n=l/20 and assume that k=0. The second resolvent identity implies for each i,j∈{0,1}

$$\begin{array}{cc} G(0,l)=\; & G_{\left[-n+i,n-j\right]}\left(0,n-j\right)G\left(n-j+1,l\right)\\ \; & +G_{\left[-n+i,n-j\right]}\left(0,-n+i\right)G\left(-n+i-1,l\right), \end{array}$$

and

$$\begin{array}{cc} G(0,l)= & G\left(0,l-n+i-1\right)G_{\left[l-n+i,l+n-j\right]}\left(l-n+i,l\right)\\ \; & +G\left(0,l+n-j+1\right)G_{\left[l-n+i,l+n-j\right]}\left(l+n-j,l\right). \end{array}$$

Thus, we obtain for $\lambda \notin \cap_{i,j\in \{0,1\}}\mathrm{Bad}\;\left(H^{\left[-n+i,n-j\right]},e^{-cn}\right)$ for any i,j∈{0,1}

$$\left|G\left(0,l\right)\right|\leq e^{-cn}\left(|G(n-j+1,l)|+|G(-n+i-1,l)|\right),$$

and for $\lambda \notin \cap_{i,j\in \{0,1\}}\mathrm{Bad}\;\left(H^{\left[l-n+i,l+n-j\right]},e^{-cn}\right)$ for any i,j∈{0,1}

$$\left|G\left(0,l\right)\right|\leq e^{-cn}\left(|G(0,l-n+i-1)|+|G(0,l+n-j+1)|\right).$$

Recall that [10]

$$Q_{I}^{\Lambda }\left(m,n\right)=\lim_{\epsilon \to 0^{+}}\frac{\epsilon }{2}\int_{I,\;I\subset \sigma (H)}{\left|G_{\lambda +i0}^{\Lambda }\left(m,n\right)\right|}^{1-\epsilon }d\lambda \leq 1,$$

where $Q_{I}^{\Lambda }$ is the eigenfunction correlator of the operator *H* restricted to a box Λ⊂Z, and $G_{\lambda +i0}^{\Lambda }$ is its resolvent operator. Therefore, we obtain

$$\begin{array}{cc} \; & \;2\int_{I,\;I\subset \sigma (H)}\frac{\epsilon }{2}{\left|G_{\lambda +i0}\left(0,l\right)\right|}^{1-\epsilon }d\lambda \\ \; & \leq 2\int_{I}e^{-(1-\epsilon )cn}\frac{\epsilon }{2}\sum_{i,j\in \{0,1\}}\left\{|G\left(n-j+1,l\right)\right|+\left|G\left(-n+i-1,l\right)\right|\\ \; & +\left|G\left(0,l-n+i-1\right)\right|+{\left|G\left(0,l+n-j+1\right)|\right\}}^{1-\epsilon }d\lambda . \end{array}$$

Since, for any α≤1, we have ${\left|a+b\right|}^{\alpha }\leq {\left|a\right|}^{\alpha }+{\left|b\right|}^{\alpha }$, we have

$$\begin{array}{cc} \; & 2\int_{I,\;I\subset \sigma (H)}\frac{\epsilon }{2}{\left|G_{\lambda +i0}\left(0,l\right)\right|}^{1-\epsilon }d\lambda \\ \; & \leq 2\int_{I}e^{-(1-\epsilon )cn}\frac{\epsilon }{2}\sum_{i,j\in \{0,1\}}{\left\{|G\left(n-j+1,l\right)\right|}^{1-\epsilon }+{\left|G\left(-n+i-1,l\right)\right|}^{1-\epsilon }\\ \; & \;+{\left|G\left(0,l-n+i-1\right)\right|}^{1-\epsilon }+{\left|G\left(0,l+n-j+1\right)\right|}^{1-\epsilon }\}d\lambda , \end{array}$$

When we take ϵ→0 and Λ↗Z, we obtain

$$\begin{array}{cc} Q_{I}\left(0,l\right) & \leq 2e^{-cn}\sum_{i,j\in \{0,1\}}\{Q_{I}\left(n-j+1,l\right)+Q_{I}\left(-n+i-1,l\right)\\ \; & +Q_{I}\left(0,l-n+i-1\right)+Q_{I}\left(0,l+n-j+1\right)\}\\ \; & \leq 16e^{-cn}\leq 16e^{-\frac{c}{20}l}\equiv 16e^{-c^{\prime }\;l}, \end{array}$$

where in the second inequality, we used the fact that $Q_{I}\left(n,m\right)\leq 1$ for any n,m∈Z, and the last one follows from the choice of *n*.

## 5. Schrödinger Operators with Potentials Generated by Hyperbolic Transformations

In this section, we will present an extension of the results of works by Avila, Damanik, and Zhang in [33,34], where the spectral localisation was established. The proof of our main result is motivated by and based on Theorem 2.10 in [34] and is an upgrade from spectral localisation to the exponential decay of the corresponding eigenfunction correlator (Theorem 5). This allows us to conclude that the corresponding entanglement entropy (7) obeys the Area Law at the bottom of the spectrum (Theorem 4).

We will use two inputs from [33,34]—the uniform positivity of the Lyapunov exponent and a uniform large deviation type estimate.

### 5.1. Necessary Facts of the Subshifts of Finite Type (SFT) and the SFT-Driven Schrödinger Operators

#### 5.1.1. Shifts of Finite Type and Corresponding Markov Chains

Let

A={1,2,…,k},k≥2,

be a finite alphabet equipped with the discrete topology. Consider the product space $A^{Z}$ whose topology is generated by the cylinder sets formed by fixing a finite set of coordinates

$$\left[n;a_{0},a_{1},\ldots ,a_{m}\right]=\left\{\omega \in A^{Z}\;|\;\omega_{n+i}=a_{i},\;0\leq i\leq m\right\}.$$

The topology is metrizable and the metric is

(83) $$d\left(\omega ,\omega \right)=0\;\mathrm{for}\;\mathrm{any}\;\omega \in A^{Z},$$

$$d\left(\omega ,\tilde{\omega }\right)=e^{-N(\omega ,\tilde{\omega })}\;\mathrm{for}\;\mathrm{any}\;\omega ,\tilde{\omega }\in A^{Z},\;\omega \neq \tilde{\omega },$$

where

$$N\left(\omega ,\tilde{\omega }\right)=\max\;\{M\geq 0\;|\;\omega_{n}={\tilde{\omega }}_{n}\;\mathrm{for}\;\mathrm{all}\;\;|n|\leq M\}.$$

Let $A={\left\{A_{ij}\right\}}_{i,j=1}^{k}$ be a k×k matrix such that $A_{ij}\in \left\{0,1\right\}$ for all 1≤i,j≤k. Introduce the two-sided shift of finite type as

$$\Omega_{A}=\left\{{\left(\omega_{n}\right)}_{n\in Z}\in A^{Z}\;|\;A_{\omega_{n}\omega_{n+1}}=1\;\mathrm{for}\;\mathrm{all}\;\;n\in Z\right\}.$$

The one-sided shift of finite type is defined in a similar way, just by replacing Z with $Z_{+}$.

The left shift map $T:\Omega_{A}\to \Omega_{A}$ is defined by ${\left(T\omega \right)}_{n}=\omega_{n+1}$. The *subshift of finite type* is a restriction of *T* to a closed *shift-invariant* subspace Ω, namely, (Ω,T) is a subshift of finite type if $\Omega \subset \Omega_{A}$ such that $T^{n}\Omega \subset \Omega$ for any n∈Z.

Let $P={\left\{P_{ij}\right\}}_{i,j=1}^{k}$ be a stochastic k×k matrix, i.e., $P_{ij}\geq 0$, for any 1≤i,j≤k and $\sum_{i=1}^{k}P_{ij}=1$ for any 1≤j≤k. We say that *P* is compatible with the above matrix *A* if $P_{ij}>0\Leftrightarrow A_{ij}=1$. Assume that *P* is irreducible, namely, that for all i,j∈{1,…,k}=A there exists an integer n≥0 such that ${\left(P^{n}\right)}_{ij}>0$. Since *P* is an irreducible stochastic matrix, the Perron–Frobenius Theorem states that there exists a unique maximal eigenvalue of *P*, λ=1, and the rest of the eigenvalues satisfy $|\lambda_{j}|<1$. Let $p=(p_{1},\ldots ,p_{k})$ be the eigenvector corresponding to the maximal eigenvalue λ=1 satisfying $p_{i}>0$ for all 1≤i≤k normalized so that $\sum_{i=1}^{k}p_{i}=1$, such that pP=p. Given vector p, we can define a probability measure $P=P_{P}$ on $\Omega_{A}$ by

(84) $$P_{P}\left(\left[0;a_{0},\ldots ,a_{n}\right]\right)=p_{a_{0}}P_{a_{0}a_{1}}P_{a_{1}a_{2}}\cdots P_{a_{n-1}a_{n}}$$

on cylinder sets. By the Kolmogorov Extension Theorem, this uniquely defines a measure on the whole σ-algebra. It is easy to check that the measure $P_{P}$ on $\Omega_{A}$ is *T*-invariant by checking that $P_{P}$ and $T_{*}P_{P}$ agree on cylinder sets where $T_{*}P_{P}$ is the pushforward measure, namely, $T_{*}P_{P}\left(B\right)=P_{P}\left(T^{-1}B\right)$ for any measurable $B\in A^{Z}$.

This measure is called a Markov measure, and it is well-known that the topological support of $P_{P}$ is a subshift of finite type $\Omega_{A}$ with the adjacency matrix *A* where $A_{ij}=1$ if and only if $P_{ij}>0$. Moreover, the measure $P_{P}$ is *T*-ergodic if and only if the matrix *P* is irreducible, and *T* has a fixed point if and only if $P_{ii}>0$ for some 1≤i≤k, which implies that *P* is aperiodic.

Let $\left(\Omega_{A},T\right)$ be a subshift of finite type defined above equipped with the σ-algebra, and let P be a probability measure on $\Omega_{A}$ that is ergodic with respect to the shift *T*. According to (Lemma 3.4 in [33]), the measure $P_{P}$ defined by (84) possesses an important property, known as the bounded distortion property and given by the following definition.

**Definition** **4.** *A positive measure ν possesses the bounded distortion property if there exists a constant C≥1 such that for all cylinders $\left[n;a_{0},\ldots ,a_{j}\right]$ and $\left[m;b_{0},\ldots ,b_{l}\right]$ in $\Omega_{A}$ where m>n+j and $\left[n;a_{0},\ldots ,a_{j}\right]\cap \left[m;b_{0},\ldots ,b_{l}\right]\neq \emptyset$, we have*

(85) $$C^{-1}\leq \frac{\nu (\left[n;a_{0},\ldots ,a_{j}\right]\cap \left[m;b_{0},\ldots ,b_{l}\right])}{\nu \left(\left[n;a_{0},\ldots ,a_{j}\right]\right)\nu \left(\left[m;b_{0},\ldots ,b_{l}\right]\right)}\leq C.$$

This notion (concept) is used in various branches of mathematics to denote objects and/or properties that do not differ much from certain “standards” of the branch. For instance, in advanced analysis (conformal mapping, complex analysis, dynamical systems) it states that the derivative of a function (or map) does not change excessively within a specific region, ensuring that the function (or map) is close enough to a similarity transformation.

In probability theory, the bounded distortion, also known as mixing, ensures that if X and Y are two events determined by sufficiently distant segments of an infinite sequence of random variables, then the joint probability P(XY) is close enough to the product P(X)P(Y) describing the independent events. In particular, in the context of Markov chains, the bounded distortion property means that the transition probability of the chain provides a sufficiently good form of the above decoupling of Markov measure P(…). For the Markov chain that arises in the proof of Theorem 4, the rate of decoupling is exponential in the distance between the events, see the proof of an important bound (105).

#### 5.1.2. Assumptions and Definitions

Here, we formulate additional definitions and results from [33,34] that we will need below.

The main object of our study in this section is the one-dimensional discrete Schrödinger operators $H_{\omega }$ (26), where the potential $V_{\omega }\left(n\right)$ is generated by the subshift of finite type $\left(\Omega_{A},T\right)$, namely, $V_{\omega }\left(n\right)=v\left(T^{n}\omega \right),\omega \in \Omega_{A}$, where $v:\Omega_{A}\to R$ is a function from one of the following two classes (see [33,34]).

**Definition** **5.** *(i) A function $v:\Omega_{A}\to R$ is said to be locally constant if there exists an integer $n_{0}\geq 0$ such that for each $\omega \in \Omega_{A}$, v(ω) depends only on the cylinder set $\left[-n_{0};\omega_{-n_{0}},\ldots \omega_{n_{0}}\right]$. Denote by LC the set of all locally constant functions $v:\Omega_{A}\to R$.*

*(ii) A function $v:\Omega_{A}\to R$ is said to be α-Hölder continuous for 0<α≤1 if*

$$\underset{\omega \neq \tilde{\omega }}{\sup}\frac{|v\left(\omega \right)-v\left(\tilde{\omega }\right)|}{d{\left(\omega ,\tilde{\omega }\right)}^{\alpha }}<\infty ,$$

*where d(·,·) is the metric on $A^{Z}$ defined by (83). Denote by $C^{\alpha }\left(\Omega_{A},R\right)$, 0<α≤1, the space of real-valued α-Hölder continuous functions. The function $v\in C^{\alpha }\left(\Omega_{A},R\right)$ is said to be globally fiber bunched if there exists $\tau_{0}>0$ such that ${∥v∥}_{\infty }<\tau_{0}$. Denote the set of all globally fiber bunched functions by SH.*

Let

$$A^{\lambda }\left(\omega \right)=\left(\begin{array}{cc} \lambda -v(\omega ) & -1\\ 1 & 0 \end{array}\right)$$

be the one-step transfer matrix corresponding to the Schrödinger operator $H_{\omega }$ of (26) with the potential generated by a subshift of finite type such that v∈LC∪SH is non-constant. For any 0≤k≤l, let

(86) $$\Phi_{k,l}^{\lambda }\left(\omega \right)=A^{\lambda }\left(T^{l-1}\omega \right)\cdots A^{\lambda }\left(T^{k}\omega \right),$$

be the corresponding (l−k)-step transfer matrix, where *T* is given in (17). Denote by

(87) $$\gamma \left(\lambda \right)=\lim_{n\to \infty }\frac{1}{n}E\{\log∥\Phi_{0,n}^{\lambda }\left(\omega \right)∥\;\}=\underset{n\geq 1}{\inf}\frac{1}{n}\log∥\Phi_{0,n}^{\lambda }\left(\omega \right)∥,$$

the Lyapunov exponent, corresponding to the operator $H_{\omega }$, where E{·} is the expectation with respect to the *T*-ergodic measure P associated with the subshift of finite type.

We have the following [33,34]:

**Definition** **6.** *We say that*

*(i) $A^{\lambda }$ has uniformly positive Lyapunov exponent (PLE) on I⊂R if*

(88) $$\underset{\lambda \in I}{\inf}\gamma \left(\lambda \right)>0.$$

*(ii) $A^{\lambda }$ has uniform large deviation type estimate (ULD) on I⊂R if for every ϵ>0, there exist constants $C_{v,\epsilon },c_{v,\epsilon }>0$, depending only on v and ϵ, such that for all λ∈I and n≥1*

(89) $$P\left\{\omega \in T\;:\left|n^{-1}\log∥\Phi_{0,n}^{\lambda }\left(\omega \right)∥-\gamma \left(\lambda \right)\right|>\epsilon \right\}<C_{v,\epsilon }e^{-c_{v,\epsilon }n}.$$

Following the notation of [33,34], we denote by $Z_{v}=\left\{\lambda \in R:\gamma \left(\lambda \right)=0\right\}$. Assume that $\left(\Omega_{A},T\right)$ is a subshift of finite type and P is a *T*-ergodic measure that has bounded distortion property (85). Suppose that *T* has a fixed point on $\Omega_{A}$, and v∈LC∪SH is non-constant. Then, it follows from (Theorem 1.3 in [33]) that $Z_{v}$ is finite and there exists a set $F_{v}⊃Z_{v}$ such that for any compact interval *J* and any η>0, we have PLE (88) on

(90) $$J_{\eta }=J\setminus B\left(F_{v},\eta \right),$$

where $B(F_{v},\eta )$ denotes the open η-neighborhood of $F_{v}$. The set $J_{\eta }$ consists of a finite number of connected compact intervals. Moreover, from (Theorem 2.10 in [34]), we conclude that under the above conditions, there exists a connected compact interval *J* such that $\sigma (H_{\omega })\subset J$, where $\sigma (H_{\omega })$ is the spectrum of the operator $H_{\omega }$, such that $A^{\lambda }$ satisfies ULD (89) on $J_{\eta }$ for all η>0.

### 5.2. Proof of Theorems 4 and 5

We start with several necessary assertions.

First, we prove the following large deviation type estimate for the Schrödinger operators with potentials generated by the subshift of finite type under the assumptions of Theorems 4 and 5.

**Lemma** **3.** *For any λ∈R and any ϵ>0, we have*

$$\begin{array}{c} P\{\exists \;k,l,\;-n\leq k\leq l\leq n\;:\;|\log∥\Phi_{k,l}^{\lambda }\left(\omega \right)∥-\left(l-k\right)\gamma \left(\lambda \right)|\geq \epsilon \;n\}\leq C_{\epsilon ,v}e^{-c_{\epsilon ,v}\;n}, \end{array}$$

*where $C_{\epsilon ,v}<\infty ,c_{\epsilon ,v}>0$, and γ(·) is the corresponding Lyapunov exponent.*

**Proof.**  Let us choose 0<δ<1 such that

$$\delta \max(\log(2+{∥v∥}_{\infty }),\gamma \left(\lambda \right))\equiv \delta \max\left(\log C_{v},\gamma \left(\lambda \right)\right)\leq \frac{\epsilon }{2}.$$

We consider two cases:

(1)|l−k|≤δnand(2)|l−k|≥δn.

First case: since for any *j*, we have $∥A^{\lambda }\left(T^{j}\omega \right)∥\leq C_{v}$, we conclude that $∥\Phi_{k,l}^{\lambda }\left(\omega \right)∥\leq C_{v}^{\left|l-k\right|}$. Therefore, since |l−k|≤δn, we obtain

(91) $$\log∥\Phi_{k,l}^{\lambda }\left(\omega \right)∥\leq |l-k|\log C_{v}\leq \delta n\log C_{v}\leq \frac{\epsilon n}{2},$$

where the last inequality follows from the choice of δ.

Second case: it is proven in (Theorem 2.10 in [34]) that for |l−k|≥δn, we have

$$P\left\{\omega \in T\;:\;|\log∥\Phi_{k,l}^{\lambda }\left(\omega \right)∥-\left(l-k\right)\gamma \left(\lambda \right)|>\frac{\epsilon \;n}{2}\right\}\leq C_{1}e^{-c_{1}\;n},$$

where $C_{1}<\infty ,c_{1}>0$ may depend only on *v* and ϵ; in other words, $A^{\lambda }$ has ULD on I⊂R. By a Borel–Cantelli type argument, we conclude that

$$\begin{array}{cc} \; & P\left\{\exists \;k,l,\;-n\leq k\leq l\leq n,\;|l-k|\geq \delta n:\;|\log∥\Phi_{k,l}^{\lambda }\left(\omega \right)∥-\left(l-k\right)\gamma \left(\lambda \right)|\leq \frac{\epsilon \;n}{2}\right\}\\ \; & \geq 1-\sum_{|l-k|\geq \delta n}C_{1}e^{-c_{1}\;n}\geq 1-C_{1}\;5n^{2}e^{-c_{1}n}\geq 1-C_{2}e^{-c_{2}n}, \end{array}$$

where $5n^{2}$ is the combinatorial factor bounding the number of ways to choose k,l from [−n,n]∩Z.

On the other hand, if |l−k|≤δn, (91) implies

$$|\log∥\Phi_{k,l}^{\lambda }\left(\omega \right)∥-\left(l-k\right)\gamma \left(\lambda \right)|\leq \frac{\epsilon n}{2}+\left|l-k\right|\gamma \left(\lambda \right)\leq \frac{\epsilon n}{2}+\delta \;n\;\gamma \left(\lambda \right)\leq \frac{\epsilon n}{2}+\frac{\epsilon n}{2}=\epsilon n,$$

where the last inequality follows from the choice of δ. □

For a given operator $H_{\omega }$ of the form (26), it is well known that for any a,b∈Z, the |b−a|-transfer matrix $\Phi_{a,b}^{\lambda }\left(\omega \right)$ of (86) satisfies

$$\Phi_{a,b}^{\lambda }\left(\omega \right)=\left(\begin{array}{cc} \det(H_{\omega }^{\left[a,b\right]}-\lambda ) & \det(H_{\omega }^{\left[a+1,b\right]}-\lambda )\\ \det(H_{\omega }^{\left[a,b-1\right]}-\lambda ) & \det(H_{\omega }^{\left[a+1,b-1\right]}-\lambda ) \end{array}\right),$$

where $H_{\omega }^{\left[a,b\right]}$ is the restriction of the operator $H_{\omega }$ to [a,b]∩Z. Using Cramer’s rule, we obtain the following identity for its resolvent operator:

(92) $$G_{\left[a,b\right]}\left(k,l\right)=\frac{\det\left(H_{\omega }^{\left[a,k-1\right]}-\lambda \right)\det\left(H_{\omega }^{\left[l+1,b\right]}-\lambda \right)}{\det(H_{\omega }^{\left[a,b\right]}-\lambda )},$$

for any a,b,k,l∈Z,a≤k≤l≤b.

Given a (2n+1)×(2n+1) matrix *M*, we denote by

(93) Res(M,ϵ)={λ∈R:dist(λ,σ(M))≤ϵ},

where σ(M) denotes the spectrum of *M*, the set of spectral parameters “close” to the spectrum of *M*.

**Lemma** **4.** *There exist $n_{0}>0$ and c,η>0 such that for any $n\geq n_{0}$*

(94) $$\begin{array}{cc} P\;\{\exists i,j\in \{0,1\}\;:\;\lambda \in & \mathrm{Bad}(H_{\omega }^{\left[-n+i,n-j\right]},e^{-cn})\cup \\ \; & \mathrm{Res}\left(H_{\omega }^{\left[-n+i,n-j\right]},e^{-\frac{c}{10}n}\right)\}\leq e^{-\eta n}, \end{array}$$

*where Bad(·,ϵ) is given by (39), and Res(·,ϵ) is given by (93).*

**Proof.**  Assume that for all k,l,−n+i≤k≤l≤n−j,i,j∈{0,1},

(95) $$|\log∥\Phi_{k,l}^{\lambda }\left(\omega \right)∥-\left(l-k\right)\gamma \left(\lambda \right)|\leq \epsilon n.$$

By Lemma 3, the inequality (95) holds always for |l−k|≤δn, and for |l−k|≥δn it holds with probability $>1-C_{2}e^{-c_{2}n}$. Since $\det(H_{\omega }^{\left[k,l\right]}-\lambda )$ is an entry of the |l−k|-transfer matrix $\Phi_{k,l}^{\lambda }\left(\omega \right)$, and $∥\Phi_{k,l}^{\lambda }\left(\omega \right)∥$ is its largest eigenvalue, (95) yields

(96) $$\log|\det\left(H_{\omega }^{\left[k,l\right]}-\lambda \right)|\leq \log∥\Phi_{k,l}^{\lambda }\left(\omega \right)∥\leq \epsilon n+|l-k|\gamma \left(\lambda \right).$$

In addition, (95) implies that

(97) $$\left(2n-i-j\right)\gamma \left(\lambda \right)-\log∥\Phi_{-n+i,n-j}^{\lambda }\left(\omega \right)∥\leq \epsilon n.$$

Since the norm of a 2×2 matrix can be bounded by four times its greatest entry, (97) implies that

(98) $$\left(2n-i-j\right)\gamma \left(\lambda \right)-\max_{i,j\in \{0,1\}}\log\left|\det\left(H_{\omega }^{\left[-n+i,n-j\right]}-\lambda \right)\right|\leq 2\epsilon n.$$

Hence, for i,j∈{0,1} satisfying (98), using (96) and the representation (92) of the corresponding resolvent operator, we obtain for any −n+i≤k≤l≤n−j

(99) $$\begin{array}{cc} |G_{\left[-n+i,n-j\right]}\left(k,l\right)| & =\frac{|\det\left(H_{\omega }^{\left[-n+i,k-1\right]}-\lambda \right)||\det\left(H_{\omega }^{\left[l+1,n-j\right]}-\lambda \right)|}{\left|\det(H_{\omega }^{\left[-n+i,n-j\right]}-\lambda )\right|}\\ \; & \leq \frac{e^{\epsilon n+(k-1+n-i)\gamma (\lambda )}e^{\epsilon n+(n-j-l-1)\gamma (\lambda )}}{e^{(2n-i-j)\gamma (\lambda )-2\epsilon n}}\leq e^{4\epsilon n-\gamma (\lambda )(l-k+2)}, \end{array}$$

where the last inequality holds because i,j∈{0,1}. Since (99) holds for any −n+i≤k≤l≤n−j, in particular for k=−n+i,l=0 and for k=0,l=n−j, we obtain

$$|G_{\left[-n+i,n-j\right]}\left(0,-n+i\right)|,|G_{\left[-n+i,n-j\right]}\left(0,n-j\right)|\leq e^{-n(\gamma (\lambda )-4\epsilon )}e^{-\gamma (\lambda )}.$$

Since we have PLE on *I*, we conclude that there exist c,η>0 such that for any $n\geq n_{0}$

(100) $$P\left\{\exists i,j\in \left\{0,1\right\}\;:\;\lambda \in \mathrm{Bad}\left(H_{\omega }^{\left[-n+i,n-j\right]},e^{-cn}\right)\right\}\leq e^{-\eta n}.$$

Since

$$∥G_{\left[-n+i,n-j\right]}∥\leq \max_{k}\sum_{l}\left|G_{\left[-n+i,n-j\right]}\left(k,l\right)\right|\leq Ce^{4\epsilon n},$$

where C>0 is some constant, we obtain

$$\mathrm{dist}\left(\lambda ,\sigma \left(H_{\omega }^{\left[-n+i,n-j\right]}\right)\right)\geq \frac{1}{C}e^{-4\epsilon n};$$

hence, $\lambda \notin \mathrm{Res}(H_{\omega }^{\left[-n+i,n-j\right]},e^{-\frac{c}{10}n})$. As before, we can conclude that

(101) $$P\left\{\exists i,j\in \left\{0,1\right\}\;:\;\lambda \in \mathrm{Res}\left(H_{\omega }^{\left[-n+i,n-j\right]},e^{-\frac{c}{10}n}\right)\right\}\leq e^{-\eta n}.$$

Combining (100) and (101), we obtain (94). □

Next, we have the following general Lemma.

**Lemma** **5.** *Let $M,\tilde{M}$ be (2n+1)×(2n+1) self-adjoint matrices, ϵ>0. If $∥M-\tilde{M}∥\leq \epsilon^{2}/100$, then*

$$\mathrm{Bad}\;\left(M,\epsilon \right)\subset \mathrm{Bad}\left(\tilde{M},\frac{\epsilon }{2}\right)\cup \mathrm{Res}\left(\tilde{M},\sqrt{\epsilon }\right).$$

**Proof.**  The second resolvent identity yields

$$\begin{array}{cc} \; & \left|{\left(M-\lambda \right)}^{-1}(0,n)\right|\\ \; & \;\leq |{\left(\tilde{M}-\lambda \right)}^{-1}(0,n)|\;+∥{\left(M-\lambda \right)}^{-1}∥∥M-\tilde{M}∥∥{\left(\tilde{M}-\lambda \right)}^{-1}∥. \end{array}$$

If $\lambda \notin \mathrm{Bad}\left(\tilde{M},\frac{\epsilon }{2}\right)\cup \mathrm{Res}\left(\tilde{M},\sqrt{\epsilon }\right)$, then

$$|{\left(\tilde{M}-\lambda \right)}^{-1}(0,n)|<\frac{\epsilon }{2}\;\mathrm{and}\;∥{\left(\tilde{M}-\lambda \right)}^{-1}∥\leq \frac{1}{\sqrt{\epsilon }},$$

and since $∥\tilde{M}-M∥\leq \epsilon^{2}/100$, we obtain

$${∥\left(M-\lambda \right)}^{-1}∥\;\leq \;\frac{1}{\sqrt{\epsilon }-\left(\epsilon^{2}/100\right)}\leq \frac{2}{\sqrt{\epsilon }},$$

and finally,

$$\begin{array}{cc} \; & |{\left(\tilde{M}-\lambda \right)}^{-1}\left(0,n\right)|\;+\;∥{\left(M-\lambda \right)}^{-1}∥∥M-\tilde{M}∥∥{\left(\tilde{M}-\lambda \right)}^{-1}∥\\ \; & \;\leq \frac{\epsilon }{2}+\frac{2}{\sqrt{\epsilon }}\frac{\epsilon^{2}}{100}\frac{1}{\sqrt{\epsilon }}\leq \epsilon . \end{array}$$

□

Now, we are ready to prove Theorems 4 and 5.

**Proof of Theorem** **4.** For a given $l\geq 100n_{0}$, we fix n=⌊l/100⌋. Let $\omega \in \Omega_{A}$ be any element of the given subshift of finite type. Then, we define $\tilde{\omega }\in \Omega_{A}$ as follows. Since the adjacency matrix *A* is irreducible and aperiodic, in every row of *A*, there is an element equal to 1; that is, for every 1≤p≤s, there exists 1≤q(p)≤s such that $A_{p,q(p)}=1$. We define

(102) $${\tilde{\omega }}_{m}=\left\{\begin{array}{ccc} \omega_{m},\;\;\;\;\;\mathrm{if}\;\;\left|m\right|\leq n, & \; & \\ q(\omega_{m-1}),\;\;\mathrm{if}\;\;m>n, & \; & \\ q(\omega_{m+1}),\;\;\mathrm{if}\;\;m<-n, & \; & \end{array}\right.$$

to have $\tilde{\omega }$ the same as the given ω from −n to *n* and then continued in the way allowed by the corresponding adjacency matrix. For a given function $v:\Omega_{A}\to R,\;v\in \mathrm{LC}\cup \mathrm{SH}$, we define

$$\tilde{v}\left(\omega \right)=v\left(\tilde{\omega }\right).$$

Now, we can introduce a new potential that is an approximation of an original potential

$${\tilde{V}}_{\omega }\left(k\right)=\tilde{v}\left(T^{k}\omega \right),$$

and the new operator ${\tilde{H}}_{\omega }=-\Delta +{\tilde{V}}_{\omega }$. Then, we have for any k∈Z,

(103) $$\begin{array}{c} |V_{\omega }\left(k\right)-{\tilde{V}}_{\omega }\left(k\right)|=|v\left(T^{k}\right)-\tilde{v}\left(T^{k}\omega \right)|\leq C\mathrm{dist}\left(T^{k}\omega ,\tilde{T^{k}\omega }\right)\leq e^{-cn}, \end{array}$$

where the last inequality follows from the definition of the metric (83), taking into account that ω and $\tilde{\omega }$ differ starting from *n* by (102).

Note that if v∈LC, then for an *n* sufficiently large, we obtain $v=\tilde{v}$; thus, the difference (103) will be identically 0. If v∈SH, then c>0 in the exponent contains 0<α≤1.

Take $\epsilon =e^{-cn/10}$, where 0<c<log2/2 is coming from Lemma 4. Then, since $∥H_{\omega }-{\tilde{H}}_{\omega }∥\leq e^{-cn}<\epsilon^{2}/100$, by applying Lemma 5 twice, we obtain for any i,j∈{0,1}

$$\begin{array}{cc} \; & \mathrm{Bad}\;(H_{\omega }^{\left[k-n+i,k+n-j\right]},\epsilon )\\ \; & \subset \mathrm{Bad}\;\left({\tilde{H}}_{\omega }^{\left[k-n+i,k+n-j\right]},\frac{\epsilon }{2}\right)\cup \mathrm{Res}\;\left({\tilde{H}}_{\omega }^{\left[k-n+i,k+n-j\right]},\sqrt{\epsilon }\right)\\ \; & \subset \mathrm{Bad}\;\left(H_{\omega }^{\left[k-n+i,k+n-j\right]},\frac{\epsilon }{4}\right)\cup \mathrm{Res}\;\left(H_{\omega }^{\left[k-n+i,k+n-j\right]},\sqrt{\frac{\epsilon }{2}}\right)\\ \; & \cup \mathrm{Res}\;({\tilde{H}}_{\omega }^{\left[k-n+i,k+n-j\right]},\sqrt{\epsilon }). \end{array}$$

Using again $∥H_{\omega }-{\tilde{H}}_{\omega }∥\leq \epsilon^{2}/100$, we obtain for any i,j∈{0,1}

$$\begin{array}{cc} \; & \mathrm{Res}\;({\tilde{H}}_{\omega }^{\left[k-n+i,k+n-j\right]},\sqrt{\epsilon })\\ \; & \subset \mathrm{Res}\;\left(H_{\omega }^{\left[k-n+i,k+n-j\right]},\sqrt{\epsilon }+\frac{\epsilon^{2}}{100}\right)\subset \left(H_{\omega }^{\left[k-n+i,k+n-j\right]},2\sqrt{\epsilon }\right). \end{array}$$

Thus, we obtain

(104) $$\begin{array}{cc} \; & P\{\exists i,j\in \left\{0,1\right\}\;:\;\lambda \in \mathrm{Bad}\;\left({\tilde{H}}_{\omega }^{\left[k-n+i,k+n-j\right]},4e^{-cn}\right)\\ \; & \;\cup \mathrm{Res}\;\left({\tilde{H}}_{\omega }^{\left[k-n+i,k+n-j\right]},e^{-cn/2}\right)\}\leq P\{\exists i,j\in \left\{0,1\right\}\;:\;\\ \; & \;\lambda \in \mathrm{Bad}\;\left(H_{\omega }^{\left[k-n+i,k+n-j\right]},e^{-cn}\right)\cup \mathrm{Res}\;\left(H_{\omega }^{\left[k-n+i,k+n-j\right]},e^{-cn/10}\right)\leq e^{-\eta n}, \end{array}$$

where the last inequality follows from Lemma 4. The entries of the resolvent corresponding to the finite operator ${\tilde{H}}_{\omega }^{\left[k-n+i,k+n-j\right]}$ are rational functions whose numerator and denominator are polynomials of degree ≤3n. Thus, for any i,j∈{0,1} and any *k*, the set

$$\begin{array}{cc} \; & {\tilde{\mathrm{Bad}}}_{k}\left({\tilde{H}}_{\omega }^{\left[k-n+i,k+n-j\right]},\epsilon \right)\\ \; & \equiv \cap_{i,j\in \{0,1\}}\left\{\mathrm{Bad}\;\left({\tilde{H}}_{\omega }^{\left[k-n+i,k+n-j\right]},\frac{\epsilon }{2}\right)\cup \mathrm{Res}\;\left({\tilde{H}}_{\omega }^{\left[k-n+i,k+n-j\right]},\sqrt{\epsilon }\right)\right\} \end{array}$$

is a union of at most 48n intervals.

Let us show that if |k−l|≥100n, then the sets ${\tilde{\mathrm{Bad}}}_{l}\left({\tilde{H}}_{\omega }^{\left[k-n+i,k+n-j\right]},\epsilon \right)$ and ${\tilde{\mathrm{Bad}}}_{l}\left({\tilde{H}}_{\omega }^{\left[k-n+i,k+n-j\right]},\epsilon \right)$ are “almost independent”, namely, that there exists $\tilde{c}>0$ such that the following exponential mixing holds:

(105) $$\begin{array}{cc} \; & |P({\tilde{\mathrm{Bad}}}_{l}\left({\tilde{H}}_{\omega }^{\left[k-n+i,k+n-j\right]},\epsilon \right)\cap {\tilde{\mathrm{Bad}}}_{l}\left({\tilde{H}}_{\omega }^{\left[l-n+i,l+n-j\right]},\epsilon \right))-\\ \; & P\left({\tilde{\mathrm{Bad}}}_{l}\left({\tilde{H}}_{\omega }^{\left[k-n+i,k+n-j\right]},\epsilon \right)\right)P\left({\tilde{\mathrm{Bad}}}_{l}\left({\tilde{H}}_{\omega }^{\left[l-n+i,l+n-j\right]}\epsilon \right)\right)|\leq e^{-\tilde{c}\left|k-l\right|}\leq e^{-100\tilde{c}n}. \end{array}$$

As we indicated in the preliminaries, there is a one-to-one correspondence between subshifts of finite type and Markov chains. To prove (105), we will pass to the corresponding Markov chain setting. Let S={1,…,k} be the phase space of the Markov chain. Since P is *T*-ergodic, we conclude that the corresponding transition matrix *P* is irreducible; hence, the Markov chain is irreducible. Since *T* has a fixed point, then *P* and, thus, the Markov chain are aperiodic, namely, there exists an integer *m* such that for all 1≤i≤k, we have ${\left(P^{m}\right)}_{ii}>0$. Thus, (105) follows from the *Convergence Theorem* (see, e.g., (Theorem 4.9 in [51])). Assume that |k−l|≥100n, $n=\lfloor \frac{l}{100}\rfloor$ and

$${\tilde{\mathrm{Bad}}}_{k}\left({\tilde{H}}_{\omega }^{\left[k-n+i,k+n-j\right]},\epsilon \right)\cap {\tilde{\mathrm{Bad}}}_{l}\left({\tilde{H}}_{\omega }^{\left[l-n+i,l+n-j\right]},\epsilon \right)\neq \emptyset .$$

Then, either one of the edges of set ${\tilde{\mathrm{Bad}}}_{k}\left({\tilde{H}}_{\omega }^{\left[k-n+i,k+n-j\right]},\epsilon \right)$ is inside ${\tilde{\mathrm{Bad}}}_{l}\left({\tilde{H}}_{\omega }^{\left[l-n+i,l+n-j\right]},\epsilon \right)$ or vice versa. Since there are at most 48n intervals in each of these sets, there are at most 96n edges in total. Therefore, we obtain

(106) $$\begin{array}{cc} \; & P\{\left(\cap_{i,j\in \{0,1\}}\mathrm{Bad}\;\left(H_{\omega }^{\left[k-n+i,k+n-j\right]},4e^{-cn}\right)\right)\\ \; & \cap \left(\cap_{i,j\in \{0,1\}}\mathrm{Bad}\;\left(H_{\omega }^{\left[l-n+i,l+n-j\right]},4e^{-cn}\right)\right)\neq \emptyset \}\\ \; & \leq P\{{\tilde{\mathrm{Bad}}}_{k}\left({\tilde{H}}_{\omega }^{\left[k-n+i,k+n-j\right]},2e^{-cn}\right)\cap {\tilde{\mathrm{Bad}}}_{l}\left({\tilde{H}}_{\omega }^{\left[l-n+i,l+n-j\right]},2e^{-cn}\right)\neq \emptyset \}\\ \; & \leq 192ne^{-\eta n}+e^{-\tilde{C}n}, \end{array}$$

where the last inequality follows from (105), (104), and the fact that the maximal number of edges in both sets is 192n. By a Borel–Cantelli type argument, we obtain

$$\begin{array}{cc} P\;\{\exists n_{0}:\;\forall n\geq n_{0}\;\; & {\tilde{\mathrm{Bad}}}_{k}\left({\tilde{H}}_{\omega }^{\left[k-n+i,k+n-j\right]},2e^{-cn}\right)\\ \; & \cap {\tilde{\mathrm{Bad}}}_{l}\left({\tilde{H}}_{\omega }^{\left[l-n+i,l+n-j\right]},2e^{-cn}\right)=\emptyset \}\\ \; & \geq 1-\sum_{n=1}^{\infty }192ne^{-n(\eta +\tilde{C})}\geq 1-Ce^{-\eta n}. \end{array}$$

Now, applying Chebyshev’s inequality, we conclude that for any $l\geq 100n_{0},$

$$\begin{array}{cc} E\{Q_{I}\left(0,l\right)\} & \leq E\{Q_{I}1_{{\tilde{\mathrm{Bad}}}_{k}\left({\tilde{H}}_{\omega }^{\left[k-n+i,k+n-j\right]},2e^{-cn}\right)\cap {\tilde{\mathrm{Bad}}}_{l}\left({\tilde{H}}_{\omega }^{\left[l-n+i,l+n-j\right]},2e^{-cn}\right)=\emptyset }\}\\ \; & +\;E\{Q_{I}\left(1-1_{{\tilde{\mathrm{Bad}}}_{k}\left({\tilde{H}}_{\omega }^{\left[k-n+i,k+n-j\right]},2e^{-cn}\right)\cap {\tilde{\mathrm{Bad}}}_{l}\left({\tilde{H}}_{\omega }^{\left[l-n+i,l+n-j\right]},2e^{-cn}\right)=\emptyset }\right)\}\\ \; & \leq 16e^{-c^{\prime }n}+192ne^{-(\eta +\tilde{C})n}\leq Ce^{-\tilde{c}l}, \end{array}$$

where the second inequality follows from Lemma 1 and (106). □

**Proof of Theorem** **5.** It follows from Theorem 4 and Criterion 2 that the corresponding entanglement entropy in this case obeys the Area Law at the bottom of the spectrum. □

**Remark** **8.** *An analogous proof can be given for half-line operators associated with one-sided shift. In particular, our proof applies to the famous doubling map.*

## 6. Conclusions

We studied rigorously the large-block asymptotic properties of the entanglement entropy of a macroscopic collection of free fermions living in $Z^{d},\;d\geq 1$. By using the reduction of this many-body problem to a highly nontrivial problem on the large distance behaviour of the Fermi projection of the one body Hamiltonian of fermions, we analyse a variety of new interesting cases where the coefficients of the Hamiltonian are either almost-periodic (quasi-periodic or limit periodic) or more complex dynamically generated ergodic processes, known as the subshifts of finite type (e.g., the Arnold cat map and the doubling map).

Basing on a variety of recent results of the spectral theory of this class of ergodic operators, including those obtained in the paper, we prove that for all considered cases, the entanglement entropy obeys the so-called area law, according to which the entropy is asymptotically proportional to the surface area of the block. This asymptotic behavior has been established in the last decade for the case where the coefficients of the one body Hamiltonian are random and its spectrum is pure point. We show that for dynamically generated ergodic coefficients, the pure point spectrum is also behind the scene, but the mathematical mechanism connecting the spectrum and the area law is quite different from that for random coefficients and is based on the property of uniform localisation of eigenfunctions (ULE), absent in random cases, valid in certain almost periodic cases, and proven in the paper for new almost periodic cases and for subshifts of finite type (d=1). Our results provide a serious support for the universality of the area law for free fermions, extending its validity to the large class of incommensurate external fields. Of course, the study of non-interacting systems is always combined with the hope that they will serve as guides for more realistic interacting ones.

## Data Availability

No new data were created or analyzed in this study. Data sharing is not applicable to this article.

## Appendix A. Another Proof of Bound (42) for the Maryland Model

We will consider a more general quantity E{(ϕ(H))(m,n)} for a bounded ϕ:R→C. We have by spectral theorem and (49)–(51)

$$\begin{array}{cc} \; & E\left\{|\left(\phi \left(H\right)\right)\left(m,n\right)|\right\}\leq \sum_{l\in Z^{d}}E\{|\phi \left(\lambda_{l}\left(\omega \right)\right)\psi_{l}\left(\omega ,m\right)\psi_{l}\left(\omega ,n\right)\left|\right\}\\ \; & =\sum_{l\in Z^{d}}\int_{T^{d}}|\phi (\lambda_{0}\left(\omega +\langle ff,l\rangle \right)||\psi_{0}\left(\lambda_{0}\left(\omega +\langle ff,l\rangle \right),m-l\right)\psi_{0}\left(\lambda_{0}\left(\omega +\langle ff,l\rangle \right),n-l\right))|d\omega \\ \; & \;=\sum_{l\in Z^{d}}\int_{T^{d}}|\phi (\lambda_{0}\left(\omega \right)|\;|\psi_{0}\left(\lambda_{0}\left(\omega \right),m-l\right)\psi_{0}\left(\lambda_{0}\left(\omega \right),n-l\right)|d\omega , \end{array}$$

and in the last equality, we use the periodicity of $\lambda_{0}:T\to R$.

Next, it follows from (52) and (53) that dω=n(λ)dλ; hence, the r.h.s. above is

$$\int_{R}\left|\phi \left(\lambda \right)\right|\sum_{l\in Z^{d}}\left|\psi_{0}\left(\lambda ,m-l\right)\psi_{0}\left(\lambda ,n-l\right)\right|n\left(\lambda \right)d\lambda .$$

Now, by using (33) and simple formulae (76)–(79), we obtain

$$E\left\{|\left(\phi \left(H\right)\right)\left(m,n\right)|\right\}\leq \tilde{C}e^{-c|m-n|/2}\int_{R}\left|\phi \left(\lambda \right)\right|n\left(\lambda \right)d\lambda .$$

In particular, using the indicator $\chi_{\left(-\infty ,\varepsilon_{F}\right]}$ as ϕ, we obtain having (52) (cf. (33))

$$E\{|P\left(m,n\right)|\}|\leq \tilde{C}e^{-\tilde{c}\left|m-n\right|}N\left(\varepsilon_{F}\right).$$

The role of C<∞ in (42) plays now $\tilde{C}N\left(\varepsilon_{F}\right)<C$ and $0<c=2\tilde{c}$.
